# Neutrophils and Ly6C^hi^ monocytes collaborate in generating an optimal cytokine response that protects against pulmonary *Legionella pneumophila* infection

**DOI:** 10.1371/journal.ppat.1006309

**Published:** 2017-04-06

**Authors:** Cierra N. Casson, Jessica L. Doerner, Alan M. Copenhaver, Jasmine Ramirez, Alicia M. Holmgren, Mark A. Boyer, Ingharan J. Siddarthan, Sara H. Rouhanifard, Arjun Raj, Sunny Shin

**Affiliations:** 1 Department of Microbiology, Perelman School of Medicine, University of Pennsylvania, Philadelphia, Pennsylvania, United States of America; 2 Department of Bioengineering, School of Engineering and Applied Science, University of Pennsylvania, Philadelphia, Pennsylvania, United States of America; Emory University School of Medicine, UNITED STATES

## Abstract

Early responses mounted by both tissue-resident and recruited innate immune cells are essential for host defense against bacterial pathogens. In particular, both neutrophils and Ly6C^hi^ monocytes are rapidly recruited to sites of infection. While neutrophils and monocytes produce bactericidal molecules, such as reactive nitrogen and oxygen species, both cell types are also capable of synthesizing overlapping sets of cytokines important for host defense. Whether neutrophils and monocytes perform redundant or non-redundant functions in the generation of anti-microbial cytokine responses remains elusive. Here, we sought to define the contributions of neutrophils and Ly6C^hi^ monocytes to cytokine production and host defense during pulmonary infection with *Legionella pneumophila*, responsible for the severe pneumonia Legionnaires’ disease. We found that both neutrophils and monocytes are critical for host defense against *L*. *pneumophila*. Both monocytes and neutrophils contribute to maximal IL-12 and IFNγ responses, and monocytes are also required for TNF production. Moreover, natural killer (NK) cells, NKT cells, and γδ T cells are sources of IFNγ, and monocytes direct IFNγ production by these cell types. Thus, neutrophils and monocytes cooperate in eliciting an optimal cytokine response that promotes effective control of bacterial infection.

## Author summary

The innate immune system is one of the first lines of defense against invading microorganisms. Many innate immune cell types, including neutrophils and Ly6C^hi^ monocytes, are rapidly recruited to sites of infection. Neutrophils and monocytes are thought to engage both overlapping and distinct effector mechanisms for antimicrobial clearance. Neutrophils are generally viewed as playing a direct bactericidal role, whereas monocytes are thought to be bactericidal, but are also considered to be the major producers of important cytokines that prime or activate subsequent responses. However, neutrophils are also capable of making many of the same cytokines produced by monocytes, but whether neutrophils and monocytes contribute to cytokine responses in a redundant or non-redundant manner remains unclear. Here, we demonstrate that neutrophils and Ly6C^hi^ monocytes contribute to the production of cytokines required for host defense against *Legionella pneumophila*, which causes the severe pneumonia Legionnaires’ disease. Both neutrophils and monocytes contribute to maximal production of the cytokines IL-12 and IFNγ, and monocytes are also required for production of the cytokine TNF. Finally, we show that monocytes are required for optimal IFNγ production by innate and innate-like lymphocytes, allowing for increased control of *L*. *pneumophila* infection. Our work demonstrates that neutrophils and monocytes cooperate in shaping a protective cytokine response that ensures successful host defense.

## Introduction

The innate immune system is essential for host defense against bacterial pathogens [1,2]. Many critical innate immune functions are carried out by a multitude of cell types, including macrophages, dendritic cells (DCs), neutrophils, and Ly6C^hi^ monocytes and their derivative cells [3]. Some of these cell types are tissue-resident, such as alveolar macrophages in the lung [4]. In contrast, Ly6C^hi^ monocytes and neutrophils exist in low numbers in the periphery during homeostasis, but are rapidly mobilized from the bone marrow and recruited to tissues early during infection [5,6]. The primary role for neutrophils in antibacterial defense is thought to involve direct bacterial killing by means of reactive oxygen species and microbicidal molecules present within granules [6–9], as well as production of neutrophil extracellular traps (NETs) [10]. Conversely, other myeloid cells, such as macrophages, DCs, and monocytes, also synthesize bactericidal molecules, but are predominantly thought to be major producers of proinflammatory cytokines, such as tumor necrosis factor (TNF), interleukin-1β (IL-1β), and IL-12 [3,5]. These cytokines orchestrate anti-bacterial effector responses that are critical for bacterial clearance. For example, Ly6C^hi^ monocytes control bacterial burdens during *Listeria monocytogenes*, *Klebsiella pneumoniae*, and *Mycobacterium tuberculosis* infection [11–14], in large part because they are an important source of IL-1β, IL-12, and IL-18 during infection and can also differentiate into DCs that produce high levels of TNF [11]. Interestingly, neutrophils can also produce TNF, IL-1β, IL-12, IL-18, IFNγ, and other cytokines in response to several bacterial and parasitic infections [15–22]. Although neutrophils and Ly6C^hi^ monocytes produce overlapping repertoires of inflammatory cytokines, it is currently unclear whether these cell types make redundant or distinct contributions to protective anti-microbial cytokine responses.

We sought to address this question in the context of pulmonary infection with the gram-negative pathogen *Legionella pneumophila*, responsible for the severe pneumonia Legionnaires’ disease [23,24]. *L*. *pneumophila* is a pathogen of freshwater amoebae and gains access to the human lung through inhalation of contaminated water aerosols [25–27]. Following uptake by alveolar macrophages, *L*. *pneumophila* replicates within these cells by deploying a Dot/Icm type IV secretion system that translocates a large repertoire of bacterial effectors that manipulate host membrane trafficking and other eukaryotic processes [28–32]. A subset of translocated effectors that block host translation elongation in combination with a host response to infection leads to a potent inhibition of global protein synthesis in infected macrophages [33–37]. Thus, infected macrophages are incapable of producing key cytokines, including TNF and IL-12, which are essential for host defense [38–41]. However, infected macrophages still synthesize and secrete the cytokines IL-1α and IL-1β [38,39], which orchestrate neutrophil recruitment to the lung [36,42–44], as well as the production of TNF and other cytokines by uninfected bystander neutrophils, Ly6C^hi^ monocytes, and DCs [38].

Although neutrophils and Ly6C^hi^ monocytes comprise the largest number of cytokine-producing cells and produce overlapping sets of cytokines during *L*. *pneumophila* infection [38,45], it is poorly understood whether these cell types make redundant or distinct contributions to the overall cytokine response. This is in part because the role of Ly6C^hi^ monocytes in cytokine production and host defense during *L*. *pneumophila* infection has been unknown. As for neutrophils, anti-Gr-1 antibody-mediated depletion suggested these cells were required for maximal IL-12 production during pulmonary *L*. *pneumophila* infection [46]. During an intravenous model of *L*. *pneumophila* infection, anti-Gr-1 antibody-based depletion suggested that neutrophils were required for IL-12 and IL-18 production and subsequent IFNγ production by natural killer (NK) cells [22]. However, the anti-Gr-1 antibodies used in these previous studies recognize an epitope common to Ly6G expressed on neutrophils and Ly6C expressed on monocytes, and anti-Gr-1 antibodies can deplete both neutrophils and Ly6C^hi^ monocytes [12,47–49], raising the question of whether Ly6C^hi^ monocytes also contribute to some of the phenotypes attributed to neutrophils. Notably, a number of mouse models of *L*. *pneumophila* infection in which neutrophil recruitment is impaired due to loss of chemokine or cytokine receptors (CXCR2 or IL-1R) [36,42–44,50] demonstrate elevated bacterial burdens, but these models can also impact recruitment or activation of other cell types [38,51].

Here, we utilized a number of complementary approaches to interrogate the relative contributions of Ly6C^hi^ monocytes and neutrophils to cytokine production and control of pulmonary *L*. *pneumophila* infection. Our data indicate that animals lacking the chemokine receptor CCR2, which is required for Ly6C^hi^ monocytes to egress from the bone marrow, exhibited a defect in both TNF and IL-12 production and monocyte-derived DC recruitment to the lung. We further found that Ly6C^hi^ monocytes and DCs produce and serve as critical sources of IL-12. Our data also demonstrate that while neutrophils are dispensable for maximal production of most inflammatory cytokines during *L*. *pneumophila* infection, they contribute to maximal IL-12 production. Intriguingly, depletion of either neutrophils or Ly6C^hi^ monocytes resulted in defective control of bacterial burdens. Furthermore, both neutrophils and Ly6C^hi^ monocytes were required for maximal production of IFNγ. NK cells and other innate-like lymphocytes in the lung served as sources of IFNγ, and monocyte-derived IL-12 directed IFNγ production by these cell types. Overall, these findings indicate important roles for both neutrophils and Ly6C^hi^ inflammatory monocytes in shaping key cytokine responses that orchestrate protective immune responses during pulmonary bacterial infection.

## Results

### Anti-Gr-1 antibody treatment depletes both neutrophils and monocytes during pulmonary infection with *L*. *pneumophila*

**Fig 1:**
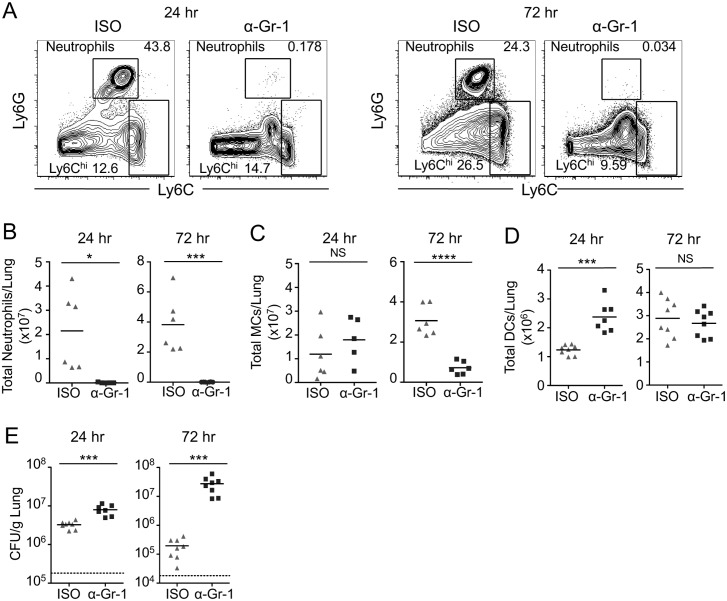
Anti-Gr-1-mediated depletion of neutrophils and Ly6C^hi^ monocytes impairs control of pulmonary *L*. *pneumophila* infection. B6 mice treated with isotype control (ISO) or anti-Gr-1 (α-Gr-1) antibody were infected with *ΔflaA L*. *pneumophila*. (A) Representative flow cytometry plots of lung cells from ISO or anti-Gr-1 treated mice at 24 hours (left) and 72 hours (right) post-infection, with gates drawn around Ly6G^hi^ neutrophils and Ly6C^hi^ cells. Ly6C^hi^ monocytes (MCs) were further defined as CD11b^+^, Ly6C^hi^ cells. Total numbers of neutrophils (B), MCs (C), and DCs (D) in the lung were quantified at 24 and 72 hours post-infection. (E) *L*. *pneumophila* CFUs in the lung were enumerated at 24 and 72 hours post-infection. Data shown are the pooled results of 2 independent experiments with 3 or 4 mice per group per experiment. * is p<0.05, *** is p<0.001 and **** is p<0.0001 by unpaired t-test. NS is not significant. Dashed line represents the limit of detection.

Mice treated with anti-Gr-1 antibodies to deplete neutrophils have a defect in bacterial clearance and IL-12 production [46], but the extent to which the anti-Gr-1 antibody might also deplete Ly6C^hi^ monocytes during pulmonary *L*. *pneumophila* infection has not previously been assessed. We therefore assayed the numbers of neutrophils and Ly6C^hi^ monocytes in WT C57BL/6 (B6) mice treated with the anti-Gr-1 antibody clone RB6-8C5. The anti-Gr-1 antibody efficiently depleted both neutrophils and Ly6C^hi^ monocytes in the lungs of naïve, uninfected mice compared to mice injected with the isotype control antibody (ISO) (S1 Fig). We then infected anti-Gr-1-treated B6 mice with Δ*flaA L*. *pneumophila*, which lacks flagellin, as this is a permissive model of infection that allows for bacterial replication in B6 mice encoding a functional NAIP5 allele [52,53]. When compared to mice injected with isotype control antibody, mice given the anti-Gr-1 antibody had significantly lower numbers of neutrophils in the lung at both 24 and 72 hours post-infection (Fig 1A and 1B). Additionally, anti-Gr-1 antibody treatment did not affect the total numbers of Ly6C^hi^ monocytes at 24 hours post-infection, but there were significantly lower numbers of Ly6C^hi^ monocytes at 72 hours post-infection (Fig 1C), similar to previous observations made during *L*. *monocytogenes* or *Toxoplasma gondii* infection, in which both neutrophils and Ly6C^hi^ monocytes were significantly depleted following anti-Gr-1 treatment [12,48]. In contrast, the total numbers of dendritic cells (DCs) did not decrease following anti-Gr-1 treatment (Fig 1D). Critically, mice that received the anti-Gr-1 antibody during infection displayed significantly higher *L*. *pneumophila* colony-forming units (CFUs) by 24 hours post-infection and a two-log increase in CFUs at 72 hours post-infection compared to isotype-treated mice (Fig 1E).

**Fig 2:**
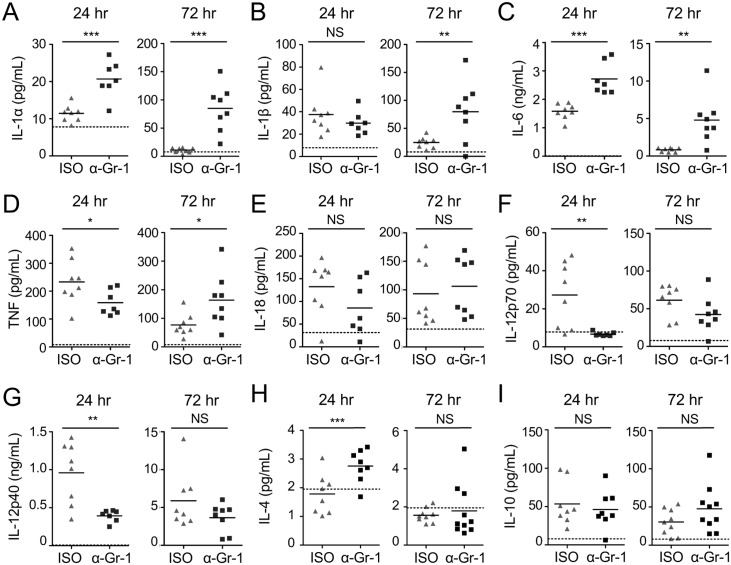
Gr-1^+^ cells are required for TNF and IL-12 production early during pulmonary infection with *L*. *pneumophila*. B6 mice treated with isotype control (ISO) or anti-Gr-1 (α-Gr-1) antibody were infected with Δ*flaA L*. *pneumophila*. Levels of IL-1α (A), IL-1β (B), IL-6 (C), TNF (D), IL-18 (E), IL-12p70 (F), IL-12p40 (G), IL-4 (H), and IL-10 (I) in the BALF at 24 and 72 hours post-infection were quantified by ELISA. Data shown are the pooled results of 2 independent experiments with 3 or 4 mice per group per experiment. * is p<0.05, ** is p<0.01, and *** is p<0.001 by unpaired t-test. NS is not significant. Dashed line represents the limit of detection.

We also measured cytokine levels in the bronchoalveolar lavage fluid (BALF) during anti-Gr-1-mediated depletion at 24 and 72 hours post-infection. We found that levels of IL-1α, IL-1β, IL-4, IL-6, IL-10, and IL-18 were unchanged or even elevated, likely reflecting increased cytokine production by other immune cell types in response to the substantial increase in bacterial load (Fig 2A–2C, 2E, 2H and 2I). In contrast, levels of TNF, the IL-12p70 heterodimer, and the IL-12p40 subunit were significantly reduced in the BALF at 24 hours post-infection in anti-Gr-1-treated mice (Fig 2D, 2F and 2G), in agreement with a previous study using anti-Gr-1-mediated depletion during pulmonary *L*. *pneumophila* infection [46]. The reduction in TNF and IL-12 is particularly notable given the increase in bacterial burden in these mice at this time (Fig 1E). Therefore, the anti-Gr-1 antibody depletes both neutrophils and monocytes, and either one or both of these cell types are critical for production of TNF, IL-12, and control of bacterial loads during pulmonary infection with *L*. *pneumophila*.

### Neutrophils are required for control of bacterial infection and maximal IL-12 production during pulmonary *L*. *pneumophila* infection

**Fig 3:**
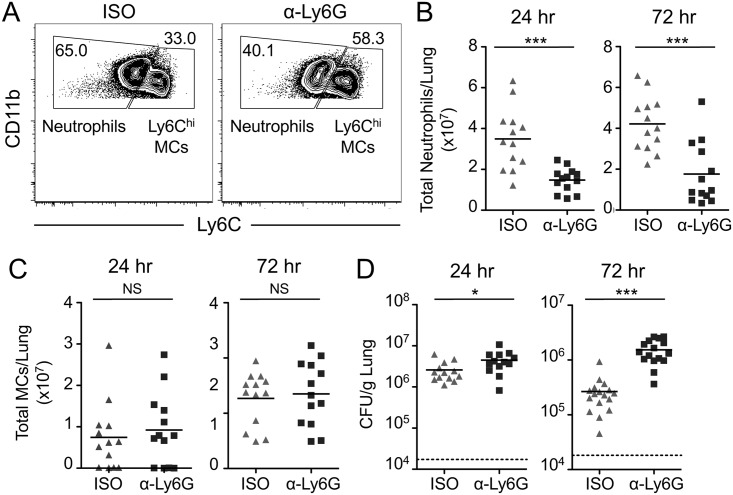
Anti-Ly6G-mediated depletion of neutrophils impairs control of pulmonary *L*. *pneumophila* infection. B6 mice treated with isotype control (ISO) or anti-Ly6G (α-Ly6G) antibody were infected with Δ*flaA L*. *pneumophila*. A) Representative flow cytometry plots of lung cells from ISO or anti-Ly6G treated mice at 24 hours post-infection, with gates drawn around neutrophils and MCs. Total numbers of neutrophils (B) and Ly6C^hi^ MCs (C) in the lung were quantified at 24 and 72 hours post-infection. (D) *L*. *pneumophila* CFUs in the lung were enumerated at 24 and 72 hours post-infection. Data shown are the pooled results of 4 independent experiments with 3 or 4 mice per group per experiment. * is p<0.05 and *** is p<0.001 by unpaired t-test. NS is not significant. Dashed line represents the limit of detection.

To interrogate the specific role of neutrophils in host protection against *L*. *pneumophila*, we employed the more selective anti-Ly6G antibody clone 1A8, which specifically depletes neutrophils and does not target monocytes [49]. The total numbers of neutrophils in the lung at both 24 and 72 hours post-infection were significantly reduced in both infected and uninfected mice treated with anti-Ly6G antibody, although the reduction was not as efficient as with anti-Gr-1 antibody (Fig 3A and 3B, and S1 Fig). The numbers of Ly6C^hi^ monocytes in the lungs of both uninfected and infected mice were unaffected by anti-Ly6G treatment, as expected (Fig 3C and S1 Fig). Importantly, this highly specific, though less robust, depletion of neutrophils resulted in significantly higher bacterial burdens in the lungs of infected mice, with a nearly one-log increase in CFUs at 72 hours post-infection (Fig 3D). Thus, our anti-Ly6G-mediated depletions demonstrate a specific role for neutrophils in host protection during pulmonary infection with *L*. *pneumophila*.

**Fig 4:**
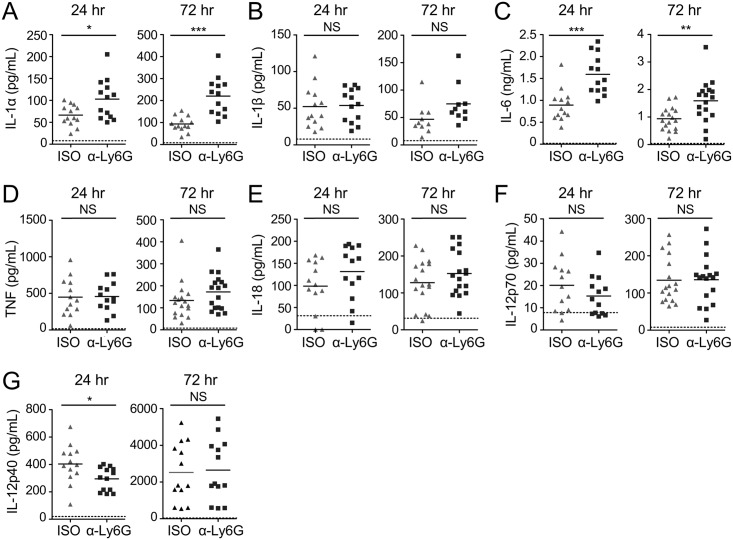
Neutrophils are required for maximal IL-12 production early during pulmonary infection with *L*. *pneumophila*. B6 mice treated with either isotype control (ISO) or anti-Ly6G (α-Ly6G) antibody were infected with *ΔflaA L*. *pneumophila*. Levels of IL-1α (A), IL-1β (B), IL-6 (C), TNF (D), IL-18 (E), IL-12p70 (F), and IL-12p40 (G) in the BALF at 24 and 72 hours post-infection were quantified by ELISA. Data shown are the pooled results of 4 independent experiments with 3 or 4 mice per group per experiment. * is p<0.05, ** is p<0.01, and *** is p<0.001 by unpaired t-test. NS is not significant. Dashed line represents the limit of detection.

The exact mechanisms underlying the contribution of neutrophils to host defense against *L*. *pneumophila* remain unclear. Neutrophils have been shown to deploy multiple effector mechanisms, such as direct microbial killing or cytokine production, during *L*. *pneumophila* infection and other infection models [6,9,10,15–21,38,45,46]. Neutrophils can produce protective cytokines, such as TNF, IL-1α, and IL-12, during pulmonary *L*. *pneumophila* infection [21,38,45,46], but the precise contribution of neutrophil-derived cytokines to the overall cytokine response is unclear. To address this question, we also measured cytokine levels in the BALF during anti-Ly6G-mediated depletion at 24 and 72 hours post-infection. Specific depletion of neutrophils with anti-Ly6G antibody did not reduce levels of other cytokines in the BALF (Fig 4A–4F), but resulted in significantly reduced levels of IL-12p40 at 24 hours post-infection (Fig 4G). Altogether, our findings delineate a specific role for neutrophils in controlling bacterial loads and early production of IL-12 during *L*. *pneumophila* lung infection [41].

### Ly6C^hi^ monocytes are protective during pulmonary infection with *L*. *pneumophila*

**Fig 5:**
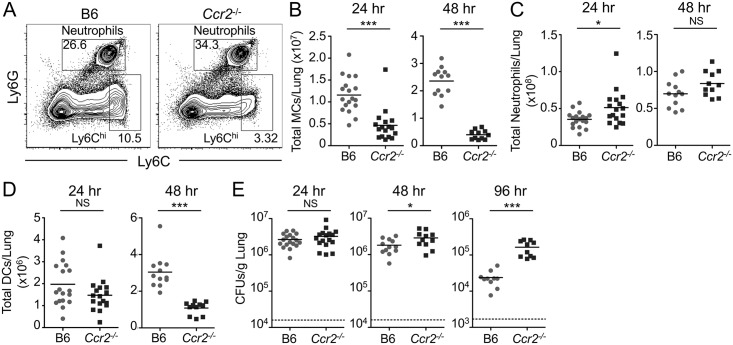
Ly6C^hi^ monocytes are required for control of pulmonary *L*. *pneumophila* infection. B6 or *Ccr2*^-/-^ mice were infected with Δ*flaA L*. *pneumophila*. (A) Representative flow cytometry plots of lung cells from B6 or *Ccr2*^-/-^ mice 24 hours post-infection, with gates drawn around neutrophils and Ly6C^hi^ cells. Ly6C^hi^ monocytes (MCs) were further defined as CD11b^+^ cells. Total numbers of Ly6C^hi^ MCs (B), neutrophils (C), and DCs (D) in the lung were quantified at 24 and 48 hours post-infection. (E) *L*. *pneumophila* CFUs in the lung were enumerated at 24, 48, and 96 hours post-infection. Data shown are the pooled results of 3 to 5 independent experiments per time point with 3 or 4 mice per group per experiment. * is p<0.05 and *** is p<0.001 by unpaired t-test. NS is not significant. Dashed line represents the limit of detection.

In addition to neutrophils, a large population of Ly6C^hi^ monocytes is also rapidly recruited into the lung during *L*. *pneumophila* infection [38]. Notably, while Ly6C^hi^ monocytes are not productively infected by *L*. *pneumophila* [21], they produce important proinflammatory cytokines, including TNF [38,45], which is required for successful control of infection [40]. We therefore sought to examine the contribution of Ly6C^hi^ monocytes to host defense against *L*. *pneumophila*. To do so, we used mice deficient for the chemokine receptor CCR2 (*Ccr2*^-/-^), because *Ccr2*^-/-^ monocytes exhibit a defect in the ability to emigrate from the bone marrow to sites of inflammation [54,55]. Indeed, following *L*. *pneumophila* infection, *Ccr2*^-/-^ mice had a significant defect in recruiting Ly6C^hi^ monocytes to the lung at 24 and 48 hours post-infection compared to B6 mice (Fig 5A and 5B). Importantly, we saw no defect in neutrophil recruitment (Fig 5C). Though *Ccr2*^-/-^ mice showed no reduction in total DC numbers in the lung at 24 hours post-infection, DCs were significantly reduced in *Ccr2*^-/-^ mice at 48 hours post-infection (Fig 5D), consistent with previous findings that Ly6C^hi^ monocytes differentiate into DCs at sites of inflammation [56]. Critically, *Ccr2*^-/-^ mice had significantly higher bacterial burdens in the lung at 48 and 96 hours post-infection (Fig 5E), demonstrating that Ly6C^hi^ monocytes play a key non-redundant role in host defense against *L*. *pneumophila*.

### Ly6C^hi^ monocytes are required for maximal TNF and IL-12 production during pulmonary *L*. *pneumophila* infection

**Fig 6:**
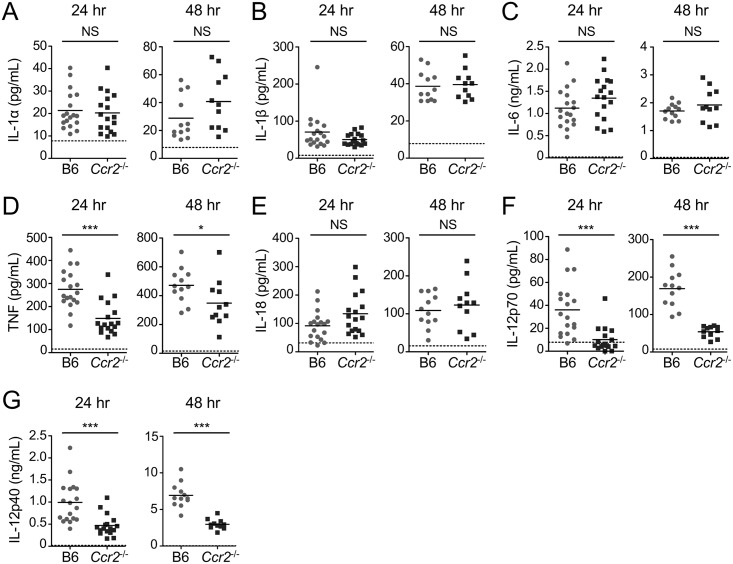
Ly6C^hi^ monocytes are required for maximal TNF and IL-12 production during pulmonary *L*. *pneumophila* infection. B6 or *Ccr2*^-/-^ mice were infected with Δ*flaA L*. *pneumophila*. Levels of IL-1α (A), IL-1β (B), IL-6 (C), TNF (D), IL-18 (E), IL-12p70 (F), and IL-12p40 (G) in the BALF at 24 and 48 hours post-infection were quantified by ELISA. Data shown are the pooled results of 3 to 5 independent experiments per time point with 3 or 4 mice per group per experiment. * is p<0.05 and *** is p<0.001 by unpaired t-test. NS is not significant. Dashed line shows the limit of detection.

Ly6C^hi^ monocytes comprise a major fraction of TNF-producing cells during pulmonary infection with *L*. *pneumophila* [38,45]. Therefore, we next asked if Ly6C^hi^ monocytes significantly contribute to the cytokine milieu during infection. We examined the levels of proinflammatory cytokines that are normally produced and secreted into the airway space during infection to determine if these cytokines are modulated in the absence of Ly6C^hi^ monocytes at 24 and 48 hours post-infection. The levels of IL-1α, IL-1β, IL-6, and IL-18 were unchanged or increased in *Ccr2*^-/-^ mice during infection, likely reflecting increased cytokine production by other immune cell types in response to the substantial increase in bacterial load (Fig 6A–6C and 6E). However, TNF levels were significantly reduced at both 24 and 48 hours post-infection in the absence of Ly6C^hi^ monocytes (Fig 6D). This finding indicates that Ly6C^hi^ monocytes are indeed a major source of TNF during pulmonary infection with *L*. *pneumophila*, consistent with previous findings showing that Ly6C^hi^ monocytes comprise the majority of TNF-producing cells during infection [38,45]. In addition to TNF, *Ccr2*^-/-^ mice also had a significant defect in IL-12p70 and IL-12p40 production at both 24 and 48 hours post-infection (Fig 6F and 6G), in agreement with recent findings [57]. The defect in IL-12 production exhibited by *Ccr2*^-/-^ mice was greater than the defect we observed in anti-Ly6G-depleted mice (Fig 4G), indicating that monocytes are more critical than neutrophils for IL-12 production. Altogether, these data demonstrate that Ly6C^hi^ monocytes are required for maximal production of the protective cytokines TNF and IL-12 during pulmonary *L*. *pneumophila* infection.

### Ly6C^hi^ monocytes, dendritic cells, and neutrophils produce IL-12 during pulmonary *L*. *pneumophila* infection

**Fig 7:**
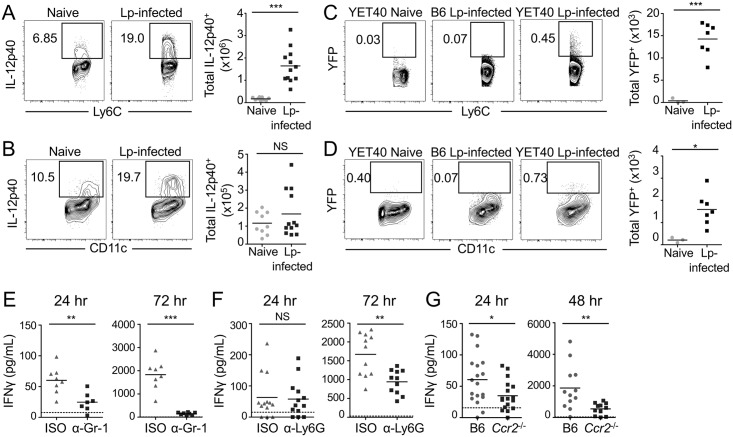
Monocytes and DCs produce IL-12, and monocytes and neutrophils are required for IFNγ production during pulmonary *L*. *pneumophila* infection. (A & B) B6 mice were uninfected (naïve) or infected with Δ*flaA L*. *pneumophila* (Lp-infected). Intracellular cytokine staining for IL-12p40 was performed on lung cells. Representative flow cytometry plots and graphs show the total numbers of IL-12p40-expressing MCs (A), and DCs (B), in the lung at 24 hours post-infection. (C & D) IL-12p40-YFP reporter mice (YET40) or B6 mice were uninfected (naïve) or infected with Lp. Representative flow cytometry plots and graphs show the total numbers of YFP-expressing Ly6C^hi^ MCs (C), and DCs (D) in the lung at 48 hours post-infection, with YFP gates drawn based on MCs and DCs from B6 mice infected with Lp. IFNγ was quantified by ELISA at 24, 48, or 72 hours post-infection in the BALF of Δ*flaA L*. *pneumophila*-infected B6 mice treated with isotype control (ISO) or anti-Gr-1 (α-Gr-1) antibody (E), infected B6 mice treated with ISO or anti-Ly6G (α-Ly6G) antibody (F), or infected B6 or *Ccr2*^-/-^ mice (G). Data shown are the pooled results of 3 (A-B), 2 (C-D), or 2 to 5 (E-G) independent experiments with 2 to 7 mice per group per experiment. * is p<0.05, ** is p<0.01 and *** is p<0.001 by unpaired t-test. NS is not significant. Dashed line shows the limit of detection.

Thus far, our data suggest that both Ly6C^hi^ monocytes and neutrophils are non-redundantly required for IL-12 production during infection. This contrasted with most of the other cytokines that we examined, suggesting that IL-12 levels are particularly sensitive to perturbation. We next sought to determine whether neutrophils and Ly6C^hi^ monocytes directly produce IL-12 during infection or whether other cell types produce IL-12 instead. In particular, Ly6C^hi^ monocytes can differentiate into DCs when they enter inflamed tissues [56], and we had observed reduced levels of DCs in the lungs of *Ccr2*^-/-^ mice following *L*. *pneumophila* infection (Fig 5D). We thus investigated whether Ly6C^hi^ monocytes or DCs produce IL-12. Compared to naïve mice, we found that in infected mice, there was a significant increase in the total numbers and percentages of Ly6C^hi^ monocytes producing IL-12 at 24 hours post-infection, as determined by flow cytometric analysis of intracellular cytokine staining (Fig 7A and S2A Fig). Though DCs also produced substantial amounts of IL-12 by intracellular cytokine staining (Fig 7B), the total numbers and percentages of IL-12-producing DCs were not significantly higher in infected mice when compared to naïve mice at 24 hours post-infection (Fig 7B and S2A Fig). As another means of tracking the cellular sources of IL-12 during infection, we used IL-12p40-YFP reporter (YET40) mice, which express IL-12p40 and YFP as a bicistronic transcript under control of the IL-12p40 promoter [58]. We found a significant increase in the percentages and numbers of monocytes and DCs that produce IL-12, as measured by YFP production at 48 hours post-infection, when compared to naïve YET40 mice (Fig 7C and 7D and S2B Fig). These data suggest that both monocytes and DCs are major sources of IL-12 during pulmonary infection, in agreement with recent findings [57].

As mice treated with anti-Ly6G antibodies had a significant defect in IL-12 production at 24 hours post-infection (Fig 4G), we next asked if neutrophils also serve as a direct source of IL-12 during pulmonary infection. We found that a significantly higher number of neutrophils in the lungs of infected mice produced IL-12 compared to naïve mice at 24 hours-post infection, as determined by intracellular cytokine staining (S2C Fig), consistent with a prior study indicating that neutrophils stain positive for IL-12 during infection with *Legionella pneumophila* [46]. However, the level of IL-12 staining observed in neutrophils was relatively modest in comparison to the robust levels of IL-12 that we observed in monocytes and DCs during infection. IL-12 in neutrophils is thought to be premade and stored in granules that are rapidly released upon infection [19], thus making it difficult to detect IL-12 by intracellular cytokine staining. Therefore, we also employed IL-12p40-YFP reporter mice as a complementary method for assessing IL-12 production by neutrophils. Infected YET40 mice had a significant increase in the percentage and number of YFP^+^ neutrophils compared to uninfected mice at 48 hours post-infection (S2D Fig), further supporting that neutrophils indeed do produce IL-12 during pulmonary infection with *L*. *pneumophila*. Additionally, we performed single-molecule RNA fluorescence *in situ* hybridization (FISH), which enables highly specific and sensitive detection of individual mRNA transcripts, as another method to assess IL-12 production in neutrophils from the lungs of infected mice. While we observed single cell variability in *Il12* mRNA expression in neutrophils, some neutrophils were found to have very high *ll12* absolute mRNA counts (ranging from 0–100 individual mRNAs per cell) (S3A and S3B Fig). Overall, these data indicate that Ly6C^hi^ monocytes, DCs, and neutrophils all serve as cellular sources of IL-12 during *L*. *pneumophila* infection.

### Ly6C^hi^ monocytes and neutrophils are required for maximal IFNγ production during pulmonary *L*. *pneumophila* infection

As our data thus far indicate that both neutrophils and Ly6C^hi^ monocytes produce IL-12 and are required for maximal IL-12 responses during pulmonary *L*. *pneumophila* infection, we next sought to determine the functional roles of neutrophil- and Ly6C^hi^ monocyte-derived IL-12 during infection. IL-12 can elicit production of the cytokine IFNγ [59,60], and IFNγ is required for host defense against pulmonary *L*. *pneumophila* infection [61]. Therefore, we next examined whether neutrophils and Ly6C^hi^ monocytes are also required for IFNγ production during *L*. *pneumophila* infection.

**Fig 8:**
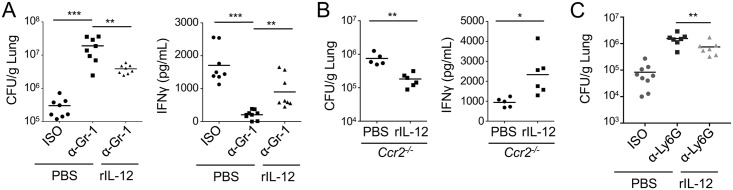
Exogenously administered IL-12 partially restores bacterial control and IFNγ production in mice lacking neutrophils and/or monocytes following infection. B6 mice treated with isotype control (ISO) or anti-Gr-1 (α-Gr-1) antibody (A), Ccr2^-/-^ mice (B), or B6 mice treated with isotype control or anti-Ly6G (α-Ly6G) antibody (C) were infected with Δ*flaA L*. *pneumophila* and given 500ng of recombinant IL-12 (rIL-12) intranasally. CFUs were enumerated and IFNγ levels in BALF were measured 72 hours post-infection. Data shown are the pooled results of 2–3 independent experiments with 3 or 4 mice per group per experiment. * is p<0.05, ** is p<0.01 and *** is p<0.001 by unpaired t-test. NS is not significant.

In mice depleted of both neutrophils and monocytes with the anti-Gr-1 antibody, we observed a significant and sustained decrease in IFNγ levels in the BALF at 24 and 72 hours post-infection (Fig 7E), in agreement with previous studies also using anti-Gr-1 antibody-mediated depletion [22,46]. In mice depleted of neutrophils with the more specific anti-Ly6G antibody, we also observed a significant decrease in IFNγ production at 72 hours post-infection compared to isotype-treated mice (Fig 7F). Because *Ccr2*^-/-^ mice also exhibited a profound defect in IL-12 production, we next examined secretion of IFNγ into the airway space of *Ccr2*^-/-^ mice during infection. Strikingly, *Ccr2*^-/-^ mice also produced significantly less IFNγ at both 24 and 48 hours post-infection when compared to WT mice (Fig 7G), in agreement with recent findings [57]. Furthermore, exogenous administration of recombinant IL-12 significantly enhanced control of bacterial loads in anti-Gr-1-treated mice, anti-Ly6G-treated mice and *Ccr2*^-/-^ mice (Fig 8A–8C). Recombinant IL-12 treatment also significantly restored IFNγ production in anti-Gr-1-treated mice and *Ccr2*^-/-^ mice (Fig 8A and 8B). Overall, these data indicate that neutrophils and monocytes are required for maximal production of both IL-12 and IFNγ, and that IL-12 from monocytes and/or neutrophils directs other cell types to produce IFNγ, thus promoting bacterial clearance.

### Ly6C^hi^ monocytes instruct NK cells and innate-like lymphocytes to produce IFNγ during pulmonary *L*. *pneumophila* infection

**Fig 9:**
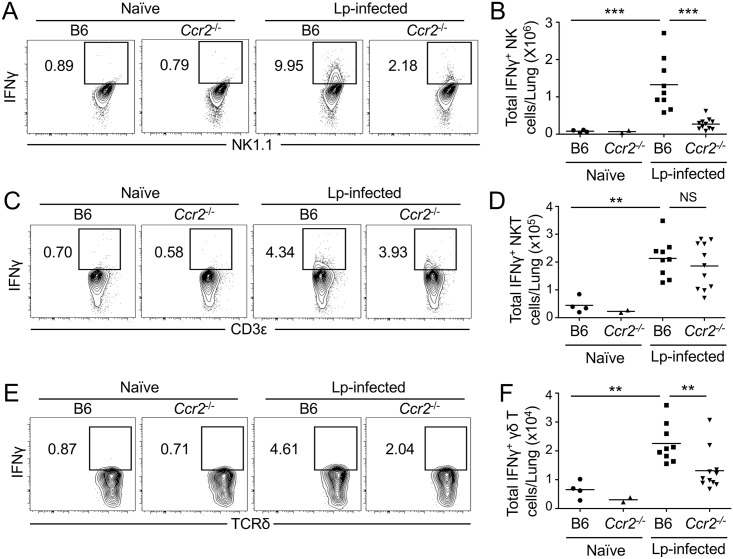
NK cells, NKT cells, and γδ T cells produce IFNγ, and monocytes are required for NK cells and γδ T cells to produce IFNγ during pulmonary *L*. *pneumophila* infection. Representative flow cytometry plots and graphs showing the total number of IFNγ^+^ NK cells (A & B), IFNγ^+^ NKT cells (C & D), and IFNγ^+^ γδ T cells (E & F) in the lungs of uninfected (naïve) or Δ*flaA L*. *pneumophila*-infected (Lp-infected) B6 or *Ccr2*^-/-^ mice at 24 hours post-infection. Data shown are the pooled results of 2 independent experiments with 2 to 7 mice per group per experiment. ** is p<0.01 and *** is p<0.001 by unpaired t-test. NS is not significant. Dashed line represents the limit of detection.

We next asked which cell types produce IFNγ in response to either neutrophil- or monocyte-dependent IL-12. NK cells produce IFNγ early during pulmonary *L*. *pneumophila* infection [61]. Thus, we first examined the impact of neutrophil or Ly6C^hi^ monocyte deficiency on IFNγ production by NK cells and focused on 24 hours post-infection, as the defect in IFNγ production was already evident at this timepoint (Fig 7E). In infected B6 mice, we observed a significant increase in the numbers and percentages of IFNγ^+^ NK cells compared to naïve B6 mice, in agreement with previous findings (Fig 9A and 9B and S4B Fig). In contrast, significantly fewer NK cells produced IFNγ in *Ccr2*^-/-^ mice (Fig 9A and 9B and S4B Fig). Though a subset of NK cells express CCR2 [62,63], we did not observe a defect in NK cell recruitment to the lung, as B6 mice and *Ccr2*^-/-^ mice had similar total numbers of lung NK cells during infection (S4A Fig). Additionally, *Ccr2*^-/-^ NK cells from infected mice robustly produced IFNγ when activated with PMA and ionomycin (P/I), indicating that *Ccr2*^*-/-*^ NK cells do not have a cell-intrinsic defect in the ability to produce IFNγ (S4C and S4D Fig).

IFNγ is critical for controlling *L*. *pneumophila* infection, but NK cells do not account for all of the IFNγ produced during infection [61]. We therefore sought to identify additional lymphocyte populations that produce IFNγ early during pulmonary infection and determine whether IFNγ production by these other cell types also relies on neutrophils and Ly6C^hi^ monocytes. We found that lung αβ T cells do not produce detectable amounts of IFNγ at 24 hours post-infection, and the absence of Ly6C^hi^ monocytes did not alter their ability to make IFNγ (S5A and S5D Fig). We next examined γδ T cells and NKT cells, as they rapidly produce IFNγ in response to other bacterial pathogens [64,65]. We observed a significant increase in the percentage and total numbers of IFNγ-producing γδ T cells and NKT cells in the lungs of infected B6 mice compared to uninfected mice (Fig 9C–9F and S6A and S6B Fig). CCR2 deficiency did not affect IFNγ production by NKT cells at 24 hours post-infection (Fig 9C and 9D and S6A Fig), but was associated with a significantly reduced percentage and total number of IFNγ^+^ γδ T cells (Fig 9E and 9F and S6B Fig). The decreased number of IFNγ^+^ γδ T cells in *Ccr2*^-/-^ mice was not due to a defect in the total number of γδ T cells, as *Ccr2*^-/-^ and B6 mice had equivalent numbers of lung γδ T cells during infection (S6C Fig). Additionally, γδ T cells from infected *Ccr2*^-/-^ mice produced IFNγ when stimulated *in vitro* with PMA and ionomycin (P/I) (S6D and S6E Fig), suggesting that CCR2 deficiency did not cause a cell-intrinsic defect in IFNγ production. These data suggest that NK cells, NKT cells and γδ T cells all produce IFNγ during pulmonary *L*. *pneumophila* infection, and that Ly6C^hi^ monocytes direct IFNγ production by these cell types, in agreement with recent findings [57].

In infected mice treated with anti-Gr-1 antibody, the total number and percentage of IFNγ-expressing NK cells was significantly reduced compared to mice treated with isotype control antibody (S4E–S4G Fig). Furthermore, anti-Gr-1 treatment led to a significant decrease in the percentage and total numbers of IFNγ^+^ NKT cells (S7A and S7B Fig), but did not affect the percentage or total numbers of IFNγ^+^ γδ T cells (S7C and S7D Fig). Specific depletion of neutrophils using anti-Ly6G antibody did not significantly affect IFNγ production by any cell type, as measured by intracellular cytokine staining (S7E Fig). It is therefore difficult to conclude whether neutrophils direct optimal IFNγ production by a particular cell type, despite our observation that neutrophil depletion decreases total IFNγ levels in the BALF (Fig 7F). We also considered the possibility that neutrophils themselves serve as a source of IFNγ during *L*. *pneumophila* infection, as neutrophils have been shown to produce IFNγ during *T*. *gondii*, *Salmonella* Typhimurium, *Streptococcus pneumoniae*, and other infections [16–18,20]. Our attempts to analyze IFNγ production by neutrophils using antibody-based methods, such as immunofluorescence microscopy, were inconclusive. We found that the anti-IFNγ antibody exhibited highly variable and non-specific staining that was comparable in neutrophils from WT infected mice or in neutrophils from IFNγ-deficient infected mice (S8A Fig). Thus, we employed single molecule RNA FISH as a more specific method for assessing IFNγ production by neutrophils. Although *Ifng* mRNA levels were variable in neutrophils from infected mice, many neutrophils exhibited high *Ifng* absolute mRNA counts (ranging from 0–100 individual mRNAs per cell) (S3A and S3B Fig), indicating that neutrophils produce IFNγ during *L*. *pneumophila* infection.

## Discussion

During pulmonary *L*. *pneumophila* infection, neutrophils and Ly6C^hi^ monocytes are both rapidly recruited to the lung and produce overlapping sets of proinflammatory cytokines. However, the individual contributions of neutrophils and monocytes to the overall cytokine response and bacterial clearance remained unclear. Using antibody-based and genetic strategies to selectively ablate neutrophils, singly or in combination with Ly6C^hi^ monocytes, our data suggest essential and non-redundant roles for both neutrophils and Ly6C^hi^ monocytes in driving cytokine responses and control of bacterial burdens. Notably, we show that Ly6C^hi^ monocytes and neutrophils are critical for host defense against *L*. *pneumophila* infection. We also find both neutrophils and monocytes contribute to IL-12 production, and monocytes are additionally required for TNF production. Furthermore, both neutrophils and monocytes are required for maximal IFNγ production, and monocyte-derived IL-12 directs IFNγ production by NK cells and innate-like NKT and γδ T lymphocytes. Therefore, our data suggest that neutrophils and Ly6C^hi^ monocytes cooperate in shaping an optimal cytokine response that contributes to successful control of *L*. *pneumophila* infection. These findings highlight the functional crosstalk required between various innate immune cell populations to generate a protective cytokine response against bacterial infection.

Ly6C^hi^ monocytes are recruited from the bone marrow into sites of infection and are appreciated as having an important role in host defense against several pathogens [11–14]. Notably, our study, along with another recently published study [57], are among the first to examine the contribution of CCR2-dependent Ly6C^hi^ monocytes and their derivative cells to control of pulmonary *L*. *pneumophila* infection. Consistent with the requirement for CCR2 in emigration of Ly6C^hi^ monocytes from the bone marrow, *Ccr2*^-/-^ mice had a severe defect in Ly6C^hi^ monocyte recruitment to the lung following *L*. *pneumophila* infection. Critically, despite robust recruitment of neutrophils to the lung of *Ccr2*^*-/-*^ animals, which was indistinguishable from WT mice, *Ccr2*^*-/-*^ mice exhibited significant defects in TNF and IL-12 production and delayed clearance of bacteria from the lung. In addition, levels of IL-12 were more substantially reduced in anti-Gr-1-treated mice and *Ccr2*^*-/-*^ mice than in anti-Ly6G-treated mice, suggesting that monocytes are the major producers of IL-12 during *L*. *pneumophila* infection. Previously, during *L*. *monocytogenes* infection, Ly6C^hi^ monocytes were shown to produce IL-18, which then drives IFNγ production by T cells and NK cells [66]. Our data indicate that during *L*. *pneumophila* infection, Ly6C^hi^ monocytes are dispensable for IL-18 production. Instead, we found that monocytes are required for IL-12 production and subsequent IFNγ production by NK cells and γδ T cells. As monocytes are also phagocytic cells that produce nitric oxide and reactive oxygen species [67], and can exert direct bactericidal activity against *L*. *pneumophila* in an IFNγ-dependent manner [57,68], it is likely these bactericidal activities, in addition to cytokine production, also contribute to control of *L*. *pneumophila* infection.

We found that *Ccr2*^-/-^ mice were not only deficient in Ly6C^hi^ monocytes but also lacked CD11c^+^MHCII^+^ DCs by 48 hours post-infection with *L*. *pneumophila*, consistent with previous findings demonstrating that Ly6C^hi^ monocytes differentiate into DCs in inflamed tissues [56]. We show here that both Ly6C^hi^ monocytes and DCs produce IL-12 during *L*. *pneumophila* infection, and we have previously found that both Ly6C^hi^ monocytes and DCs also produce TNF [38]. Thus, absence of both cell types in the *Ccr2*^*-/-*^ mice may be responsible for the defect in TNF and IL-12 observed in these mice. As total DC numbers in the *Ccr2*^-/-^ mice were not significantly different than those observed in B6 mice at 24 hours post-infection, but were significantly different at 48 hours post-infection, it is likely that monocytes are the major producers of TNF and IL-12 at 24 hours post-infection, with an additional contribution from DCs occurring at later timepoints. Our findings are in agreement with a recent study that also identified an essential role for Ly6C^hi^ monocytes in host defense against *L*. *pneumophila* [57]. They similarly observed that Ly6C^hi^ monocytes were a critical source of IL-12 and were required for optimal IFNγ production by NK cells, NKT cells, and γδ T cells. They also found that monocytes instructed memory αβ T cells to produce IFNγ [57], whereas we did not observe robust IFNγ production by αβ T cells. This likely reflects a difference in the timepoints analyzed in the two different studies, as we analyzed an earlier timepoint (day 1) post-infection, whereas they examined day 2 post-infection, which likely allowed for increased IFNγ production by these cells.

In addition, our study clarifies the role of neutrophils in host defense against pulmonary *L*. *pneumophila* infection. The anti-Gr-1 antibody clone RB6-8C5, previously used to deplete neutrophils during pulmonary *L*. *pneumophila* infection [46], is now known to bind and deplete cells expressing either Ly6G or Ly6C, which are expressed by neutrophils, monocytes, and activated T cells [47]. Comparing Gr-1-mediated depletion to neutrophil-specific Ly6G-mediated depletion has revealed that Ly6C^hi^ monocytes, rather than neutrophils, are required for host defense against *L*. *monocytogenes* [12] and *T*. *gondii* [48]. Thus, we chose to compare anti-Gr-1-mediated depletion and anti-Ly6G-mediated depletion in the current study to examine the effects of anti-Gr-1 depletion on Ly6C^hi^ monocytes and to elucidate the role of neutrophils in host defense against *L*. *pneumophila*. Our data revealed that anti-Gr-1-mediated depletion with high doses of RB6-8C5 antibody led to a decrease in total Ly6C^hi^ monocyte numbers in the lung following *L*. *pneumophila* infection, similar to what we found in uninfected mice administered the anti-Gr-1 antibody. We found that depletion of Ly6C^hi^ monocytes was significant at 72 hours post-infection, but there was minimal depletion at 24 hours post infection. Although we did not observe depletion of monocytes at the 24 hour timepoint, we cannot rule out the possibility that the anti-Gr-1 antibody affects the function of these remaining monocytes.

Anti-Gr-1 antibody treatment was more efficient at removing neutrophils than the more neutrophil-specific anti-Ly6G antibody, but also depleted monocytes. We observed increased bacterial burdens and defective IL-12 and IFNγ production in anti-Ly6G-depleted mice, indicating that neutrophils contribute to production of IL-12 and IFNγ and control of bacterial loads in the lung. Given that neutrophils are required for maximal IL-12 and IFNγ production in infected mice, we asked whether neutrophils directly produce these cytokines. Our flow cytometry data suggest that neutrophils produce IL-12, albeit at low levels relative to monocytes and DCs. Our attempts to further determine whether neutrophils produce IL-12 and IFNγ using immunofluorescence microscopy were inconclusive, as the anti-IL-12 and anti-IFNγ antibodies resulted in comparable staining in neutrophils from WT mice or IL-12- or IFNγ-deficient mice, leading us to conclude that anti-IL-12 and anti-IFNγ staining was nonspecific under our experimental conditions (S8A and S8B Fig). However, single molecule RNA FISH, which is a more specific and sensitive method for assessing gene expression, revealed that some neutrophils from infected mice exhibited high *Il12p40* and *Ifng* absolute mRNA counts (ranging from 0–100 individual mRNAs per cell) (S3A and S3B Fig). Although the flow cytometry and RNA FISH data suggest that the majority of neutrophils do not produce high levels of IL-12 and IFNγ during *L pneumophila* infection, the large numbers (2x10^7^-4x10^7^) of neutrophils infiltrating the lung may enable these cells to significantly contribute to overall cytokine production. Neutrophils are classically thought to control bacterial burdens through direct anti-microbial mechanisms involving phagocytosis and degradation of microbes or production of NETs [6–10]. During *L*. *pneumophila* infection, neutrophils are major producers of reactive oxygen species, which is critical for effective control of infection [45]. Thus, in addition to contributing to cytokine production, neutrophils carry out important bactericidal functions that also contribute to control of *L*. *pneumophila* infection.

Our data shed additional light on the cell types involved in production of IFNγ, which is critical for control of *L*. *pneumophila* infection [57,61]. We found that NK, NKT, and γδ T cells serve as cellular sources of IFNγ, in agreement with recent findings [57]. IFNγ produced by NKT and γδ T cells is likely to be functionally important, and presumably accounts for the published finding that NK cell depletion had no impact on control of pulmonary *L*. *pneumophila* infection, despite reducing IFNγ levels [61]. IL-18 is required for maximal IFNγ production by NK cells during *L*. *pneumophila* infection [61] [65], but the critical cellular sources of IL-18 during pulmonary infection remain unknown. A previous study using an intravenous model of *L*. *pneumophila* infection found that neutrophil-derived IL-18 was critical for IFNγ production by NK cells [22]. In contrast, during pulmonary infection, our data suggest that neutrophils are not an essential source of IL-18, suggesting one or more cell types in the lung act as a redundant or critical source of IL-18. Instead, both neutrophils and monocytes were required for maximal IL-12 production, and monocytes were required for NK cells to produce IFNγ. Our data support a model in which IL-12 and IL-18 act in concert to elicit optimal IFNγ production, consistent with previous findings showing that both IL-12 and IL-18 are critical for maximal IFNγ responses [41,61,69]. Although our data suggest that neutrophils are required for maximal IFNγ production, we were unable to determine whether neutrophils direct IFNγ production by NK cells and innate-like lymphocytes. This may be due to inefficient depletion of neutrophils following anti-Ly6G treatment. Intriguingly, our data suggest that neutrophils express high levels of *Ifng* mRNA, suggesting that neutrophils themselves may serve as a source of IFNγ, as has been observed in other infection models [16,20,70].

Overall, our findings provide new insight into the roles of Ly6C^hi^ monocytes and neutrophils during pulmonary *L*. *pneumophila* infection. We find that both monocytes and neutrophils are critical for control of bacterial infection. We further show that both neutrophils and monocytes were required for maximal IL-12 and IFNγ production, but monocytes were distinct in their essential contribution to TNF production. Finally, our data demonstrate that Ly6C^hi^ monocytes instruct NK cells and innate-like lymphocytes to produce IFNγ, a key cytokine required for eventual control of *L*. *pneumophila* infection. Thus, our findings reveal critical but nuanced roles for neutrophils and Ly6C^hi^ monocytes in shaping an optimal cytokine response that ensures successful host defense against pulmonary bacterial infection.

## Materials and methods

### Ethics statement

All animal studies were performed in compliance with the federal regulations set forth in the Animal Welfare Act (AWA), the recommendations in the Guide for the Care and Use of Laboratory Animals of the National Institutes of Health, and the guidelines of the University of Pennsylvania Institutional Animal Use and Care Committee. All protocols used in this study were approved by the Institutional Animal Care and Use Committee at the University of Pennsylvania (protocols #804714 and #804928).

### Mice

C57BL/6J, B6.129S7-*Ifng*^*tm1Ts*^/J (*Ifng*^-/-^), and B6.129S1-*Il12b*^*tm1Jm*^/J (*Il12p40*^-/-^) mice were purchased from The Jackson Laboratory. *Ccr2*^-/-^ mice [54], YET40 mice [58], and *Il12p40*^-/-^ mice were purchased from The Jackson Laboratory and maintained and bred in a specific pathogen-free facility at the University of Pennsylvania.

### Bacterial strains

All experiments used the *Legionella pneumophila* serogroup 1 JR32-derived (*rpsL* and *hsdR)* strain lacking FlaA (Δ*flaA* Lp) [52,71]. *L*. *pneumophila* was cultured on charcoal yeast extract (CYE) agar plates containing streptomycin for 48 h at 37°C before infection.

### *In vivo* infections

8–12 week old mice were anesthetized by intraperitoneal (i.p.) injection of a ketamine/xylazine solution (100mg/kg ketamine and 10mg/kg xylazine) diluted in PBS. Mice were then infected intranasally (i.n.) with 40μl of 1x10^6^ bacteria suspended in PBS by instilling 10μl at a time into the nostrils. For experiments involving addition of recombinant IL-12 (Peprotech), bacteria were instilled into the nostrils 10μl at a time and 500 ng of IL-12 or PBS was administered in between doses of bacteria (alternating 20μl bacteria, 20μl cytokine, 20μl bacteria). To collect BALF, 1mL cold PBS was slowly instilled into the lung through a catheter (Jelco) and retrieved immediately. To quantify CFUs, lungs were excised, weighed, and a portion was mechanically homogenized in sterile distilled H_2_O with a gentleMACS dissociator (Miltenyi Biotec). Lung homogenates were plated on CYE plates containing streptomycin, and CFUs were enumerated.

### Antibody-mediated cell depletions

For anti-Gr-1-mediated depletions, mice were injected i.p. with 250μg of either α-Gr-1 (clone RB6-8C5) or rat IgG2b isotype control antibody (clone LTF-2) (Bio X cell). Mice were injected 16 hours before infection and then again 48 hours post-infection. For anti-Ly6G-mediated depletions, mice were injected i.p. with 250μg of either α-Ly6G (clone 1A8) or rat IgG2a isotype control antibody (clone 2A3) (Bio X Cell). Mice were injected 48 hours prior to infection and then again every 24 hours until the completion of the infection. 24 or 72 hours post-infection, lungs were harvested and CFUs were enumerated. The efficiency of neutrophil or monocyte depletion was monitored by flow cytometry.

### Flow cytometry

A portion of the lung was weighed, cut into small pieces, and digested in PBS + 5% (vol/vol) FBS, 20 U/mL DNase I (Roche) and 200–300 U/mL collagenase type IV (Worthington Biochemical) at 37°C for 40 minutes, with shaking every 5 minutes. The tissue was then mechanically homogenized with a gentleMACS dissociator (Miltenyi Biotec) and red blood cells (RBCs) were lysed with RBC lysis buffer (7.44g/L NH_4_Cl and 2.06g/L Tris-HCl, pH 7.2 in distilled H_2_O), followed by quenching with cold PBS. After filtering through a 40μM cell strainer, cells were first treated with the Zombie Yellow fixable cell viability kit (BioLegend) at room temperature in PBS + 2mM EDTA for 15–20 minutes and then stained at 4°C in PBS + 2mM EDTA, 2% BSA, 0.1% sodium azide, 5% normal rat serum, and 5% normal mouse serum (Jackson ImmunoResearch) for 40 minutes with antibodies specific for the cell surface antigens CD45 (eBioscience, clone 30-F11, e650NC), CD11c (BioLegend, clone N418, BV785), Ly6G (BioLegend, clone 1A8, PE-Cy7), Ly6C (BioLegend, clone HK1.4, APC-Cy7), Gr-1 (eBioscience, clone RB6-8C5, PE-Cy5 or APC), CD11b (BioLegend, clone M1/70, PacBlue), MHCII (BioLegend, clone M5/114.15.2, AF700), Siglec F (BD Biosciences, clone E50-2440, PE or PE-Texas Red), NK1.1 (BioLegend, clone PK136, PE-cy5), CD3ε (BD Biosciences, clone 145-2C11, PE-Texas Red), TCRβ (BioLegend, clone H57-597, APC-Cy7), and TCRδ (BioLegend, clone GL3, PE-Cy7). Data were collected on an LSR II flow cytometer (BD Biosciences) and post-collection data were analyzed using FlowJo (Treestar). Cells were always pre-gated on live, CD45^+^ singlet cells. Neutrophils were identified as live, CD45^+^, Ly6G^+^, Ly6C^int^ cells. Ly6C^hi^ monocytes were identified as live, CD45^+^, Ly6G^lo^, Ly6C^hi^, CD11b^+^ cells; in some experiments where indicated in the figure legend, an exclusion gate for B, T, and NK cells (CD19^+^, CD3^+^, NK1.1^+^) was also applied (S1B Fig). DCs were identified as live, CD45^+^, Ly6G^-^, Ly6C^-^, SiglecF^-^, CD11c^+^, MHCII^+^ cells. αβ T cells were identified as live, CD45^+^, NK1.1^-^, CD3ε^+^, TCRδ^-^, TCRβ^+^ cells. NK cells were identified as live, CD45^+^, CD3ε^-^, NK1.1^+^ cells. γδ T cells were defined as live, CD45^+^, NK1.1^-^, CD3ε^+^, TCRβ^-^, TCRδ^+^ cells. NKT cells were defined as live, CD45^+^, CD3ε^+^, NK1.1^+^ cells.

### Intracellular cytokine staining

Cells from the lung were obtained as described above and were resuspended in RPMI + 10% (vol/vol) heat-inactivated FBS, 2 mM L-glutamine, 100 IU/mL penicillin, 100 μg/mL streptomycin, and 3.3 μg/mL brefeldin A for 3.5 hours (for IFNγ ICS) or 6 hours (for IL-12 ICS) at 37°C. Cells were then washed and stained for surface markers as above. After extracellular staining, cells were fixed and permeabilized using the Cytofix/Cytoperm buffer set (BD Biosciences) and stained in Perm/Wash buffer (BD Biosciences) at 4°C for IFNγ (eBiosciences, clone XMG1.2, APC) or IL-12p40 (eBiosciences, clone C17.8, eFluor660) for 1 hour. Flow cytometric data acquisition and analysis was performed as above.

### Single-molecule RNA FISH

Single-molecule RNA FISH was performed as described previously [72]. Briefly, cells were fixed in 3.7% (vol/vol) formaldehyde in 1X PBS for 10 min at room temperature and stored in 70% ethanol at 4°C until imaging. Pools of fluorescently labelled Stellaris Custom RNA FISH probes *(Ifng*–Quasar 570; *Il12p40* –CAL Fluor Red 610, *Gapdh*–ATTO 488) (Biosearch Technologies) were hybridized to samples, followed by DAPI staining and wash steps performed in suspension. Samples were cytospun onto slides for imaging on a Nikon Ti-E inverted fluorescence microscope. For image processing, boundaries of cells were manually segmented from brightfield images and RNA spots were localized using custom software written in MATLAB [73].

### Immunofluorescence microscopy

Harvested BALF from infected WT, *Ifng*^-/-^, and *Il12p40*^*-/-*^ mice were collected and incubated in RPMI + 10% heat-inactivated FBS, 2 mM L-glutamine, 100 IU/mL penicillin, 100 μg/mL streptomycin, and 3.3 μg/mL brefeldin A for 3 hours. For anti-IFNγ staining, following brefeldin A treatment, cells were then cytospun onto Superfrost Plus microscopy slides (Thermo Fisher Scientific) and fixed in 4% paraformaldehyde, followed by washes with PBS + 0.2% Triton X-100 and blocking in PBS + 20% horse serum. Anti-mouse IFNγ antibody (eBioscience, clone XMG1.2, AlexaFluor 488) was applied in PBS + 2% horse serum + 0.1% Triton X-100, followed by washing and coverslip mounting with ProLong Gold Antifade with DAPI (Thermo Fisher Scientific). For anti-IL-12 staining, following brefeldin A treatment, cells were stained using the protocol described above for intracellular cytokine staining with anti-mouse IL-12p40 antibody (eBioscience, clone C17.8, AlexaFluor 488). Cells were then cytospun onto Fisher Scientific Superfrost Plus microscopy slides, followed by washing and coverslip mounting with ProLong Gold Antifade with DAPI (Thermo Fisher Scientific). Images were acquired on a Leica DM6000 microscope.

### ELISA

Harvested BALF from infected mice was assayed using kits specific for murine IL-1α (Biolegend), IL-1β (Biolegend), IL-6 (Biolegend), TNF (Biolegend), IL-18 (MBL International), IL-12p70 (Biolegend), IFNγ (BD Biosciences), IL-4 (Biolegend), IL-10 (Biolegend), or paired capture and detection antibodies specific for IL-12p40 (BD Biosciences).

### Statistical analysis

Plotting of all data and statistical analyses were performed using GraphPad Prism software. For comparisons between more than two groups, statistical significance was determined using a one-way ANOVA with Tukey post-test. For comparisons between two groups, statistical significance was determined using an unpaired Student’s t-test. Differences were considered statistically significant if the *P* value was <0.05.

## Supporting information

S1 Fig Anti-Gr-1 antibody treatment depletes both Ly6C^hi^ MCs and neutrophils in uninfected mice, and flow cytometric gating strategy for identifying Ly6C^hi^ MCs and neutrophils. (A) B6 mice were mock treated with PBS or treated with either isotype control antibody for anti-Gr-1 (ISO[1]), anti-Gr-1 antibody, isotype control antibody for anti-Ly6G (ISO[2]), or anti-Ly6G antibody for 1–3 days. Total numbers of neutrophils and MCs in the lung were quantified by flow cytometry. (B) Representative gating strategy for identifying Ly6C^hi^ MCs and neutrophils in the anti-Gr-1 depletion experiments shown in Fig 1, with incorporation of a lineage-specific dump gate to eliminate CD3^+^, NK1.1^+^, or CD19^+^ cells. Cells negative for CD3, NK1.1, and CD19 were further analyzed to identify Ly6G^+^Ly6C^int^ neutrophils and Ly6G^lo^Ly6C^hi^CD11b^+^ monocytes. Data shown are the results of 3 or 4 mice per condition. * is p<0.05, ** is p<0.01, and *** is p<0.001 by one-way ANOVA. NS is not significant.
(PDF)

S2 Fig IL-12-producing MCs, DCs, and neutrophils increase during pulmonary infection with *L*. *pneumophila*. (A) B6 mice were uninfected (naïve) or infected with Δ*flaA* Lp. The percentages of IL-12p40^+^ MCs and DCs in the lung were quantified at 24 hours post-infection by flow cytometry. (B) IL-12p40-YFP reporter mice (YET40) were uninfected (naïve) or infected with Δ*flaA* Lp. The percentages of YFP^+^ MCs and DCs in the lung were quantified at 48 hours post-infection. B6 mice were uninfected (naïve) or infected with Δ*flaA L*. *pneumophila* (Lp). Intracellular cytokine staining for IL-12p40 was performed on lung cells. Representative flow cytometry plots and graphs show the total numbers and percentages of IL-12p40-expressing neutrophils (C) in the lung at 24 hours post-infection. (D) IL-12p40-YFP reporter mice (YET40) were uninfected (naïve) or infected with Lp. Representative flow cytometry plots and graphs show the total numbers and percentages of YFP-expressing neutrophils in the lung at 48 hours post-infection. YFP gates were drawn based on neutrophils from B6 mice infected with Lp. Data shown are the pooled results of 3 (A & C) or 2 (B & D) independent experiments with 3 or 4 infected mice per group per experiment. * is p<0.05, ** is p<0.01, and *** is p<0.001 by unpaired t-test. NS is not significant.
(PDF)

S3 Fig Neutrophils express *Ifng* and *Il12p40* mRNA during pulmonary *L*. *pneumophila* infection. B6 mice were infected with Δ*flaA L*. *pneumophila* and RNA FISH was performed on lung cells 48 hours post-infection. Neutrophils were identified by polymorphonuclear morphology in the DAPI channel, and analysis of RNA FISH probes was performed on neutrophils (*Ifng*—Quasar 570, *Il12p40*—CAL Fluor Red 610, *Gapdh*—ATTO 488). (A) Representative images of neutrophils in each channel and histograms showing the frequency of mRNA counts are shown. (B) Graphs depict averages of absolute mRNA counts from the neutrophils of individual mice (n = 4, number of neutrophils analyzed per mouse: mouse 1 = 54, mouse 2 = 21, mouse 3 = 28, mouse 4 = 26).
(PDF)

S4 Fig IFNγ production by NK cells decreases in the absence of MCs or anti-Gr-1 treatment during pulmonary *L*. *pneumophila* infection. Graphs showing the total numbers of NK cells (A) and percentages of IFNγ^+^ NK cells (B) in the lungs of Δ*flaA* Lp-infected B6 or *Ccr2*^-/-^ mice at 24 hours post-infection. Representative flow cytometry plots (C) and graph (D) showing the percentages of IFNγ^+^ NK cells in the lungs of infected B6 mice or *Ccr2*^-/-^ mice at 24 hours post-infection, or NK cells from infected *Ccr2*^-/-^ mice treated with PMA and ionomycin (P/I). Representative flow cytometry plots (E) and graphs showing the total numbers (F) and percentages (G) of IFNγ^+^ NK cells in the lungs of naïve B6 mice or B6 mice treated with isotype control (ISO) or anti-Gr-1 antibody. Data shown are the pooled results of 2 independent experiments with 4 to 7 mice per group per experiment (A-C) or 2 to 5 independent experiments with 3 or 4 mice per group per experiment (E-G). NS is not significant, * is p<0.05, and ** is p<0.01 by unpaired t-test (A, D, E) or one-way ANOVA (B). [This figure complements Fig 9A & 9B and is derived from the same sets of experiments. Please note that the data points for the % of IFNγ^+^ NK cells in infected B6 and *Ccr2*^-/-^ mice in (B) are the same data points shown in (D).]
(PDF)

S5 Fig αβ T cells do not produce IFNγ early during pulmonary *L*. *pneumophila* infection. Representative flow cytometry plots (A) and graphs (B) showing the percentages and total numbers of IFNγ^+^ αβ T cells in the lungs of B6 or *Ccr2*^-/-^ mice that were infected with Δ*flaA* Lp or uninfected (naïve) at 24 hours post-infection. Representative flow cytometry plots (C) and graphs (D) showing the percentages and total numbers of IFNγ^+^ αβ T cells in the lungs of Δ*flaA* Lp-infected B6 mice treated with isotype control (ISO) or anti-Gr-1 (α-Gr-1) antibody at 24 hours post-infection. Data shown are the pooled results of 2 independent experiments with 4 to 7 mice per group per experiment (A & B) or the pooled results of 3 independent experiments with 3 or 4 mice per group per experiment (C & D). NS is not significant by one-way ANOVA (B) or unpaired t-test (D).
(PDF)

S6 Fig NKT cells and γδ T cells produce IFNγ following pulmonary *L*. *pneumophila* infection, and MCs are required for IFNγ production by γδ T cells but not NKT cells. Graphs showing the percentages of IFNγ^+^ NKT cells (A) and IFNγ^+^ γδ T cells (B) in the lungs of naïve and Δ*flaA* Lp-infected B6 and *Ccr2*^-/-^ mice 24 hours post-infection. (C) Total numbers of γδ T cells in the lung were quantified at 24 hours post-infection. (D) Representative flow cytometry plots and (E) graph showing the percentages of IFNγ^+^ γδ T cells in the lungs of infected B6 mice or *Ccr2*^-/-^ mice, or γδ T cells from infected *Ccr2*^-/-^ mice treated with PMA and ionomycin (P/I). Data shown are the pooled results of 2 independent experiments with 4 to 7 mice per group per experiment. * is p<0.05, ** is p<0.01, and *** is p<0.001 by unpaired t-test or one way ANOVA. NS is not significant. [This figure complements Fig 9C–9F and is derived from the same sets of experiments. Please note that the data points for the % of IFNγ^+^ γδ T cells in infected B6 and *Ccr2*^-/-^ mice in (B) are the same data points shown in (E).]
(PDF)

S7 Fig Anti-Gr-1 treatment decreases IFNγ production by NKT cells but not γδ T cells, whereas anti-Ly6G treatment does not affect IFNγ production by NK, NKT, and γδ T cells during pulmonary *L*. *pneumophila* infection. Representative flow cytometry plots and graphs showing the percentages and total numbers of IFNγ^+^ NKT cells (A and B) or IFNγ^+^ γδ T cells (C and D) in the lungs of uninfected (naïve) B6 mice or Δ*flaA* Lp-infected B6 mice treated with isotype control (ISO) or anti-Gr-1 (α-Gr-1) antibody at 24 hours post-infection. (E) Graphs showing the total numbers of IFNγ^+^ T cells, NK cells and NKT cells in the lungs of Δ*flaA* Lp-infected B6 mice treated with either isotype control antibody (ISO) or anti-Ly6G (α-Ly6G) antibody, as determined by flow cytometry. Data shown are the pooled results of 3 independent experiments with 3 or 4 mice per group per experiment (A-D) or 2 independent experiments with 3 mice per group per experiment (E). * is p<0.05 by unpaired t-test. NS is not significant.
(PDF)

S8 Fig Immunofluorescence microscopy reveals non-specific IFNγ and IL-12 staining in neutrophils from *L*. pneumophila-infected mice. WT, *Ifng*^*-/-*^ and *Il12p40*^*-/-*^ mice were infected with Δ*flaA L*. *pneumophila* (Lp) and immunofluorescence microscopy analysis was performed on neutrophils harvested by BAL at 48 hours post-infection stained with anti-IFNγ or anti-IL-12 antibodies directly conjugated to AlexaFluor488. (A) Representative images of IFNγ immunofluorescence (40x). (B) Representative images of IL-12 immunofluorescence (20x). Shown are the merged DAPI and AlexaFluor488 channels. In each image, a representative cell with positive fluorescence signal is outlined in a yellow box and displayed in a magnified inset.
(PDF)
