# Supplementary figures and images for: Neutrophils and Ly6Chi monocytes collaborate in generating an optimal cytokine response that protects against pulmonary Legionella pneumophila infection
Source: PLoS Pathog. 2017 Apr 6;13(4):e1006309. doi: 10.1371/journal.ppat.1006309 (PMC5404877; doi:10.1371/journal.ppat.1006309)

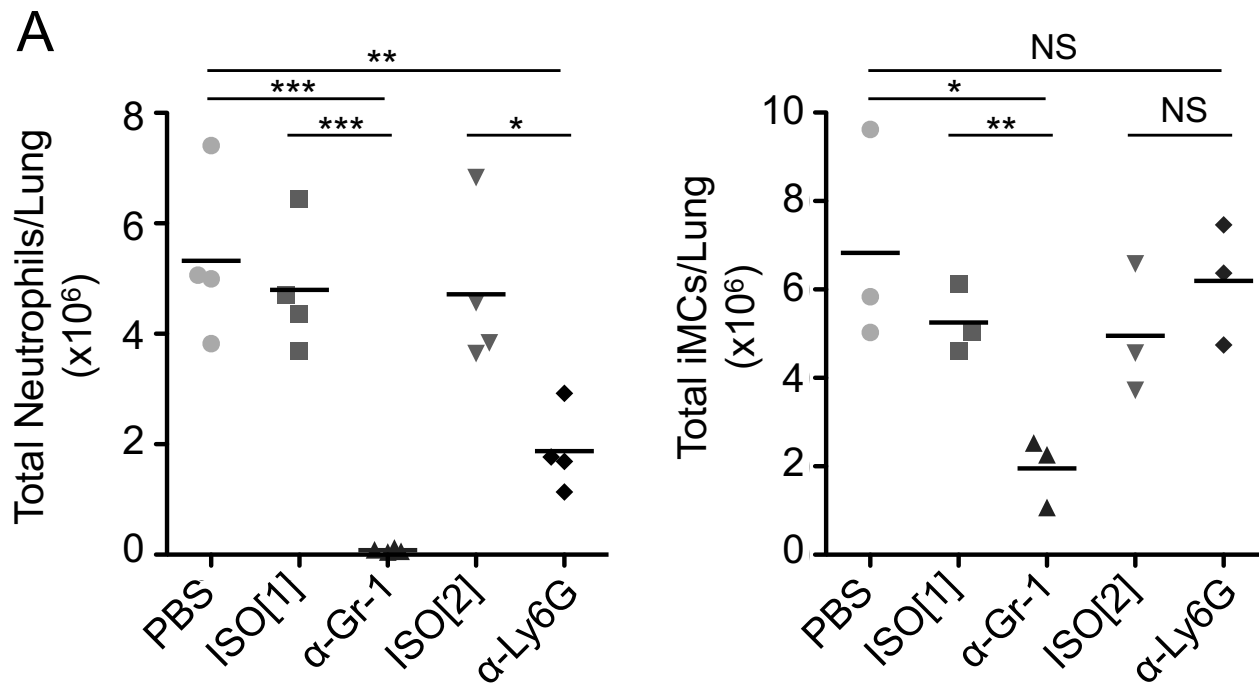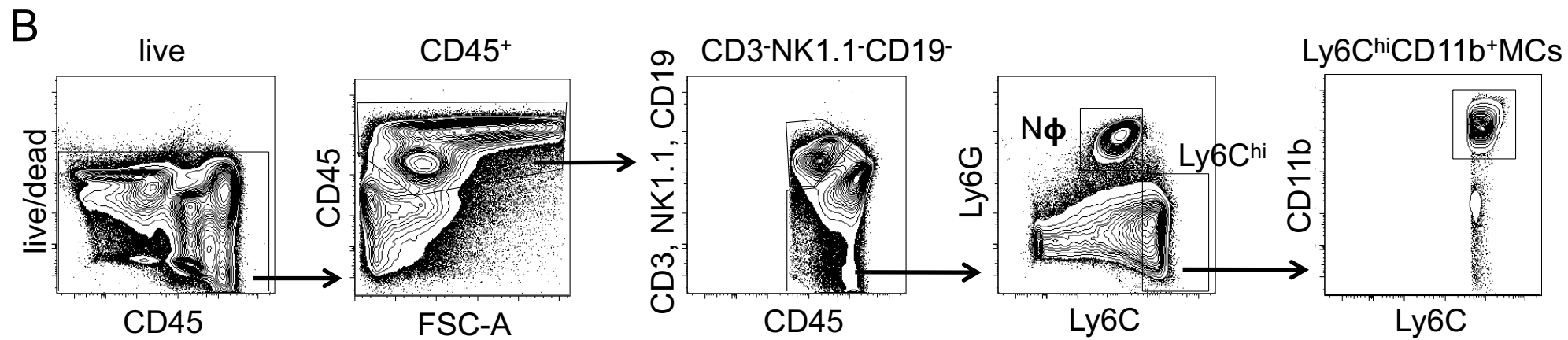

Supplement: S1 Fig — (A) B6 mice were mock treated with PBS or treated with either isotype control antibody for anti-Gr-1 (ISO[1]), anti-Gr-1 antibody, isotype control antibody for anti-Ly6G (ISO[2]), or anti-Ly6G antibody for 1–3 days. Total numbers of neutrophils and MCs in the lung were quantified by flow cytometry. (B) Representative gating strategy for identifying Ly6Chi MCs and neutrophils in the anti-Gr-1 depletion experiments shown in Fig 1, with incorporation of a lineage-specific dump gate to eliminate CD3+, NK1.1+, or CD19+ cells. Cells negative for CD3, NK1.1, and CD19 were further analyzed to identify Ly6G+Ly6Cint neutrophils and Ly6GloLy6ChiCD11b+ monocytes. Data shown are the results of 3 or 4 mice per condition. * is p<0.05, ** is p<0.01, and *** is p<0.001 by one-way ANOVA. NS is not significant. (PDF) [file ppat.1006309.s001.pdf]

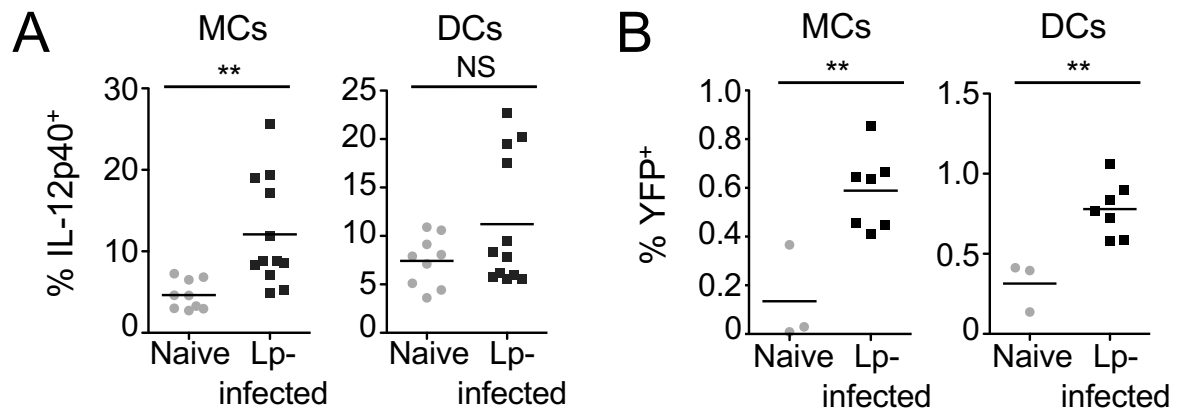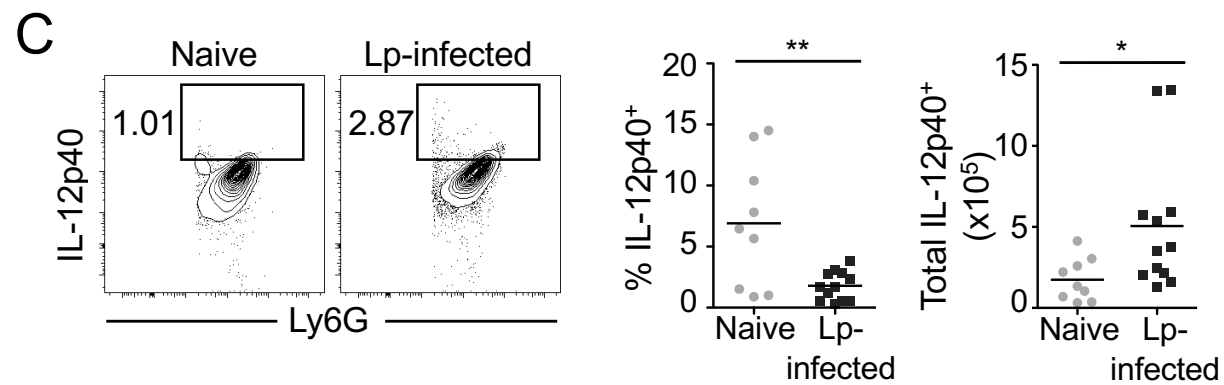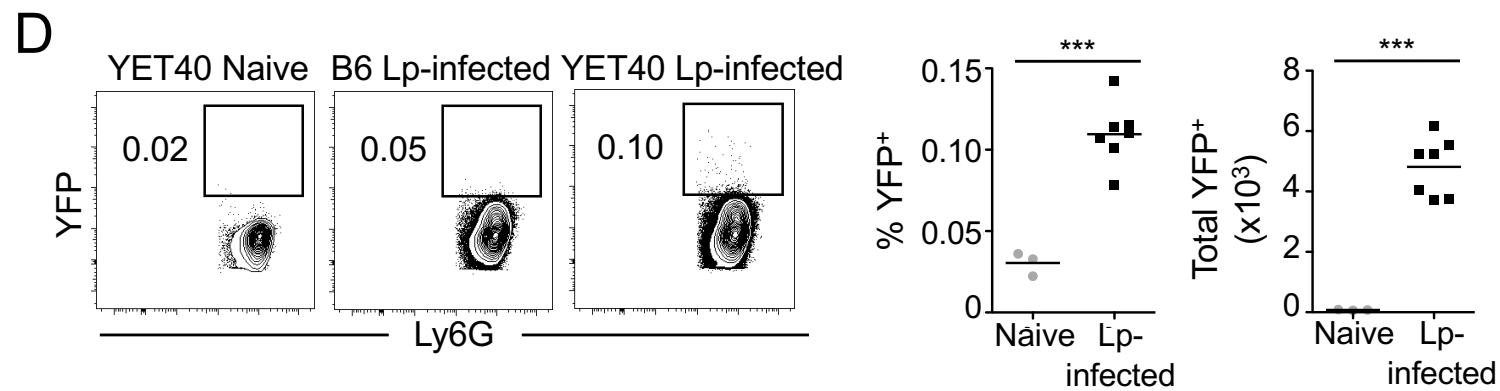

Supplement: S2 Fig — (A) B6 mice were uninfected (naïve) or infected with ΔflaA Lp. The percentages of IL-12p40+ MCs and DCs in the lung were quantified at 24 hours post-infection by flow cytometry. (B) IL-12p40-YFP reporter mice (YET40) were uninfected (naïve) or infected with ΔflaA Lp. The percentages of YFP+ MCs and DCs in the lung were quantified at 48 hours post-infection. B6 mice were uninfected (naïve) or infected with ΔflaA L. pneumophila (Lp). Intracellular cytokine staining for IL-12p40 was performed on lung cells. Representative flow cytometry plots and graphs show the total numbers and percentages of IL-12p40-expressing neutrophils (C) in the lung at 24 hours post-infection. (D) IL-12p40-YFP reporter mice (YET40) were uninfected (naïve) or infected with Lp. Representative flow cytometry plots and graphs show the total numbers and percentages of YFP-expressing neutrophils in the lung at 48 hours post-infection. YFP gates were drawn based on neutrophils from B6 mice infected with Lp. Data shown are the pooled results of 3 (A & C) or 2 (B & D) independent experiments with 3 or 4 infected mice per group per experiment. * is p<0.05, ** is p<0.01, and *** is p<0.001 by unpaired t-test. NS is not significant. (PDF) [file ppat.1006309.s002.pdf]

A

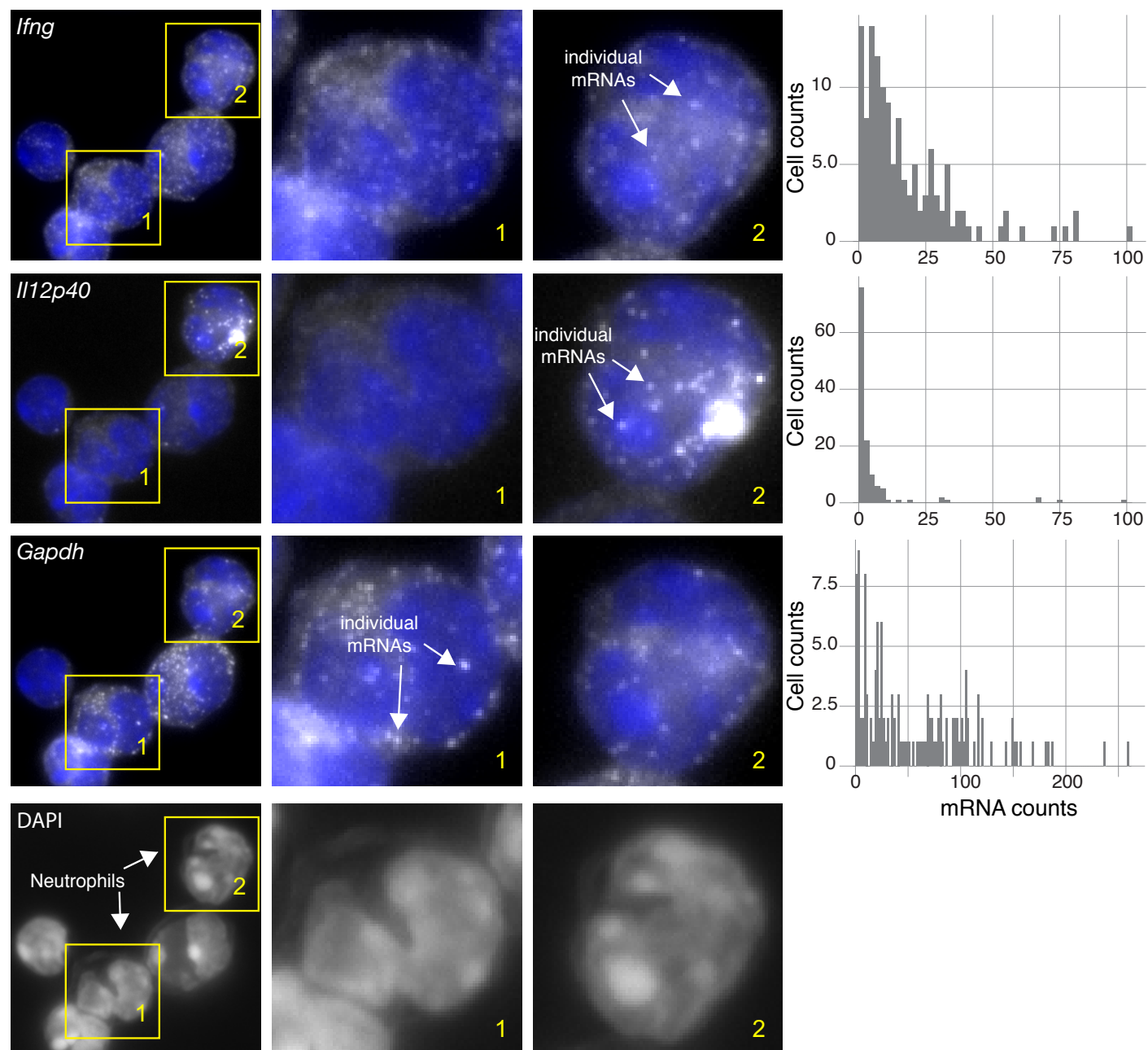

B

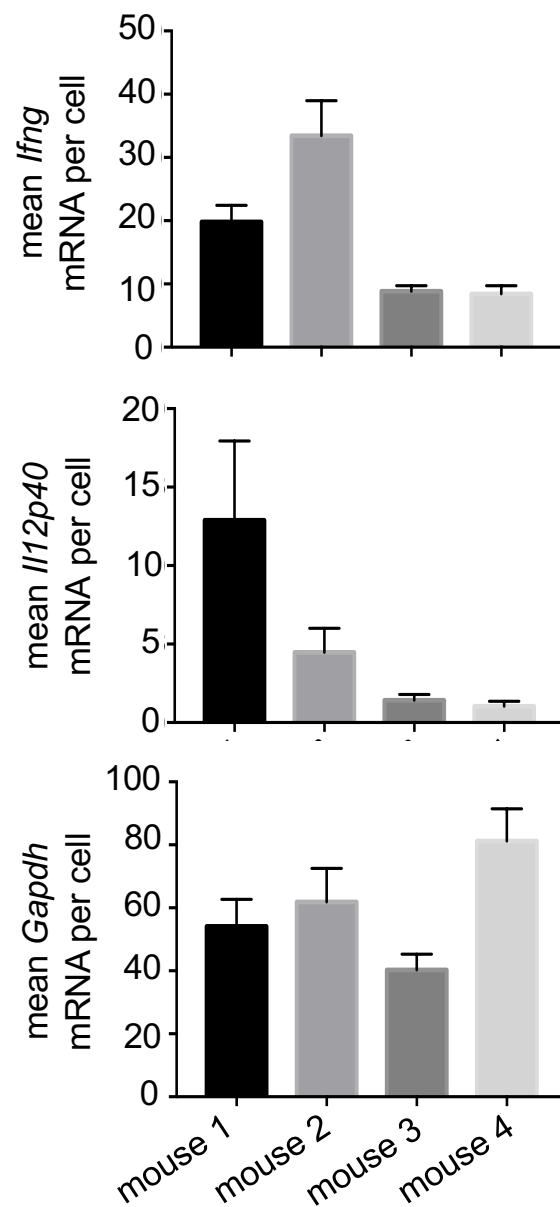

Supplement: S3 Fig — B6 mice were infected with ΔflaA L. pneumophila and RNA FISH was performed on lung cells 48 hours post-infection. Neutrophils were identified by polymorphonuclear morphology in the DAPI channel, and analysis of RNA FISH probes was performed on neutrophils (Ifng—Quasar 570, Il12p40—CAL Fluor Red 610, Gapdh—ATTO 488). (A) Representative images of neutrophils in each channel and histograms showing the frequency of mRNA counts are shown. (B) Graphs depict averages of absolute mRNA counts from the neutrophils of individual mice (n = 4, number of neutrophils analyzed per mouse: mouse 1 = 54, mouse 2 = 21, mouse 3 = 28, mouse 4 = 26). (PDF) [file ppat.1006309.s003.pdf]

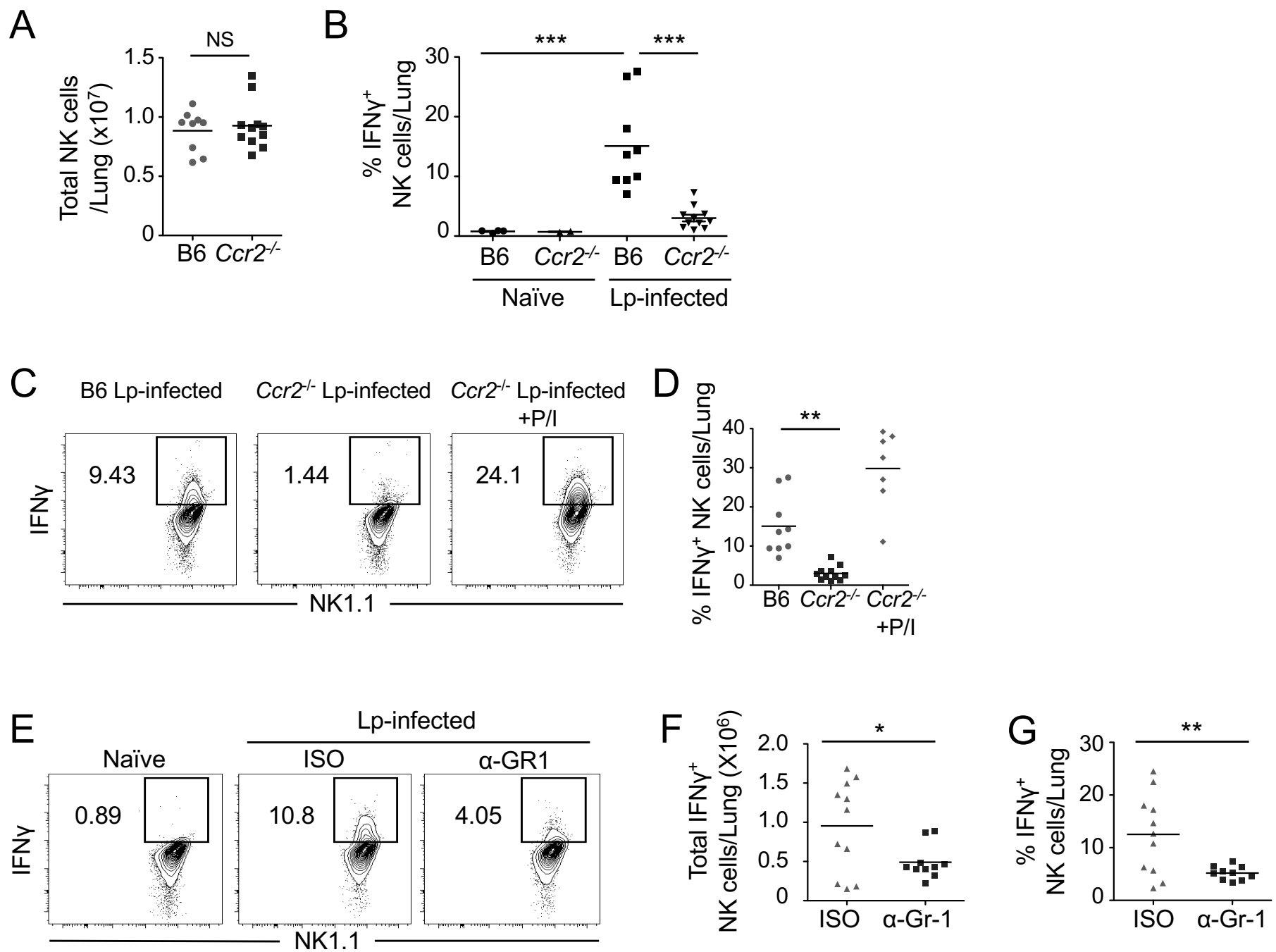

Supplement: S4 Fig — Graphs showing the total numbers of NK cells (A) and percentages of IFNγ+ NK cells (B) in the lungs of ΔflaA Lp-infected B6 or Ccr2-/- mice at 24 hours post-infection. Representative flow cytometry plots (C) and graph (D) showing the percentages of IFNγ+ NK cells in the lungs of infected B6 mice or Ccr2-/- mice at 24 hours post-infection, or NK cells from infected Ccr2-/- mice treated with PMA and ionomycin (P/I). Representative flow cytometry plots (E) and graphs showing the total numbers (F) and percentages (G) of IFNγ+ NK cells in the lungs of naïve B6 mice or B6 mice treated with isotype control (ISO) or anti-Gr-1 antibody. Data shown are the pooled results of 2 independent experiments with 4 to 7 mice per group per experiment (A-C) or 2 to 5 independent experiments with 3 or 4 mice per group per experiment (E-G). NS is not significant, * is p<0.05, and ** is p<0.01 by unpaired t-test (A, D, E) or one-way ANOVA (B). [This figure complements Fig 9A & 9B and is derived from the same sets of experiments. Please note that the data points for the % of IFNγ+ NK cells in infected B6 and Ccr2-/- mice in (B) are the same data points shown in (D).] (PDF) [file ppat.1006309.s004.pdf]

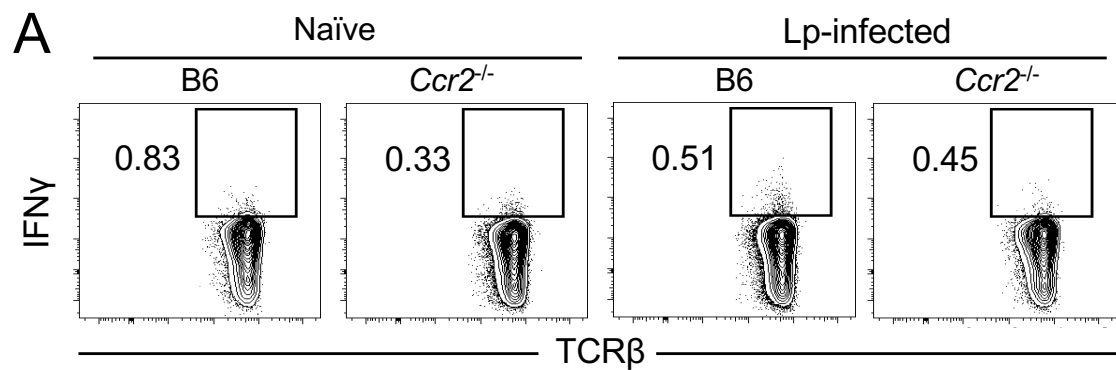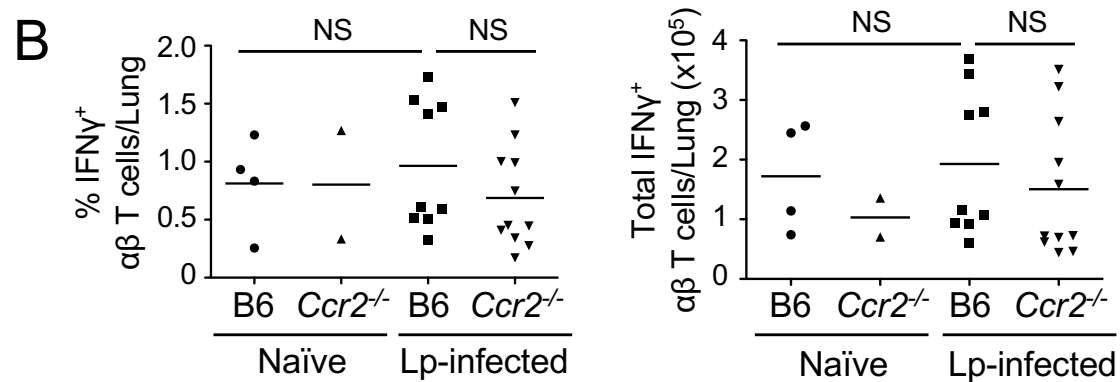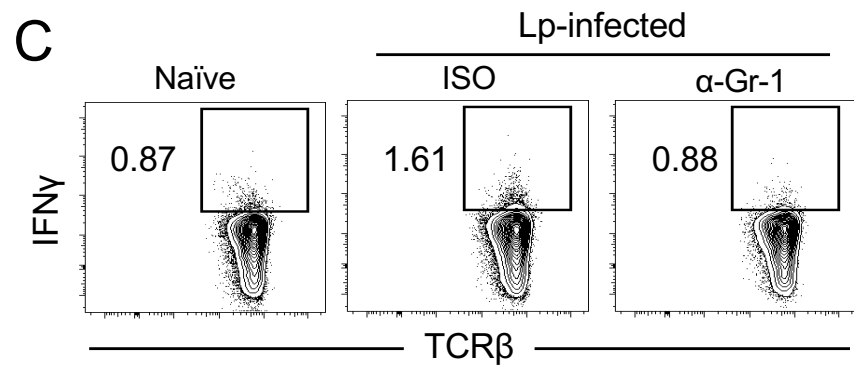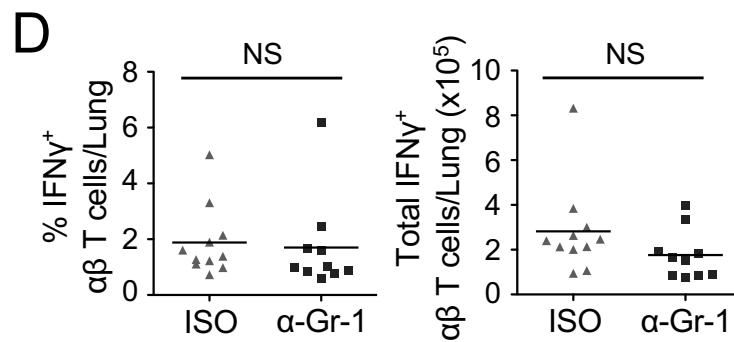

Supplement: S5 Fig — Representative flow cytometry plots (A) and graphs (B) showing the percentages and total numbers of IFNγ+ αβ T cells in the lungs of B6 or Ccr2-/- mice that were infected with ΔflaA Lp or uninfected (naïve) at 24 hours post-infection. Representative flow cytometry plots (C) and graphs (D) showing the percentages and total numbers of IFNγ+ αβ T cells in the lungs of ΔflaA Lp-infected B6 mice treated with isotype control (ISO) or anti-Gr-1 (α-Gr-1) antibody at 24 hours post-infection. Data shown are the pooled results of 2 independent experiments with 4 to 7 mice per group per experiment (A & B) or the pooled results of 3 independent experiments with 3 or 4 mice per group per experiment (C & D). NS is not significant by one-way ANOVA (B) or unpaired t-test (D). (PDF) [file ppat.1006309.s005.pdf]

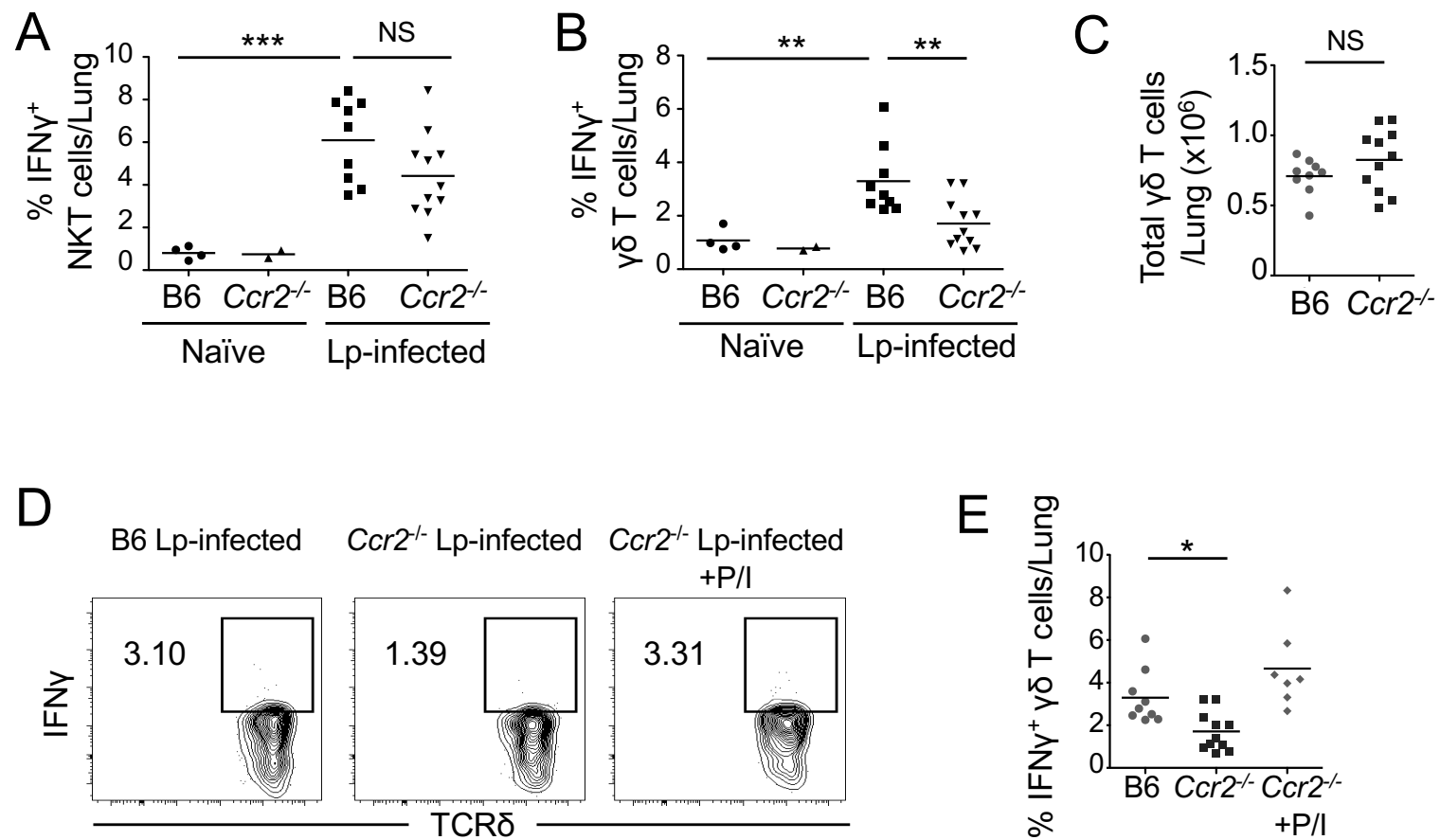

Supplement: S6 Fig — Graphs showing the percentages of IFNγ+ NKT cells (A) and IFNγ+ γδ T cells (B) in the lungs of naïve and ΔflaA Lp-infected B6 and Ccr2-/- mice 24 hours post-infection. (C) Total numbers of γδ T cells in the lung were quantified at 24 hours post-infection. (D) Representative flow cytometry plots and (E) graph showing the percentages of IFNγ+ γδ T cells in the lungs of infected B6 mice or Ccr2-/- mice, or γδ T cells from infected Ccr2-/- mice treated with PMA and ionomycin (P/I). Data shown are the pooled results of 2 independent experiments with 4 to 7 mice per group per experiment. * is p<0.05, ** is p<0.01, and *** is p<0.001 by unpaired t-test or one way ANOVA. NS is not significant. [This figure complements Fig 9C–9F and is derived from the same sets of experiments. Please note that the data points for the % of IFNγ+ γδ T cells in infected B6 and Ccr2-/- mice in (B) are the same data points shown in (E).] (PDF) [file ppat.1006309.s006.pdf]

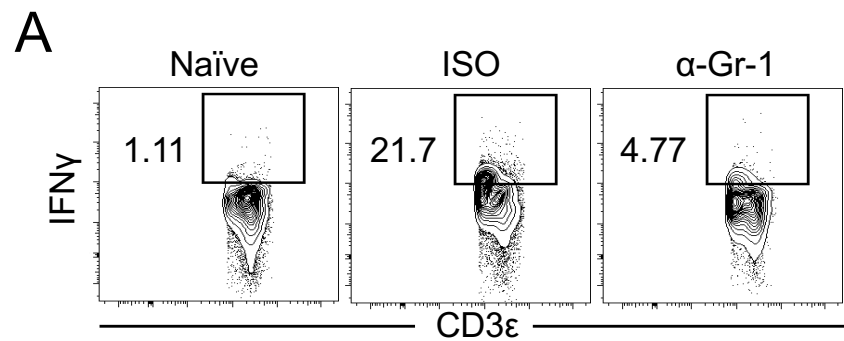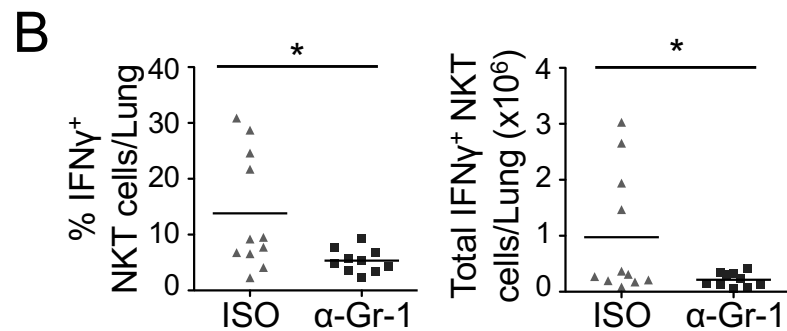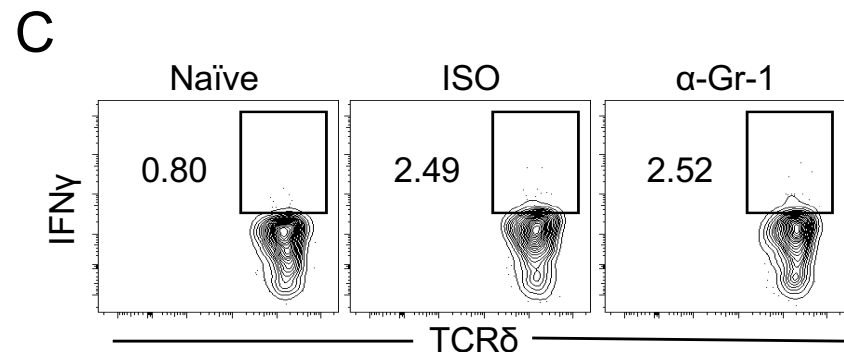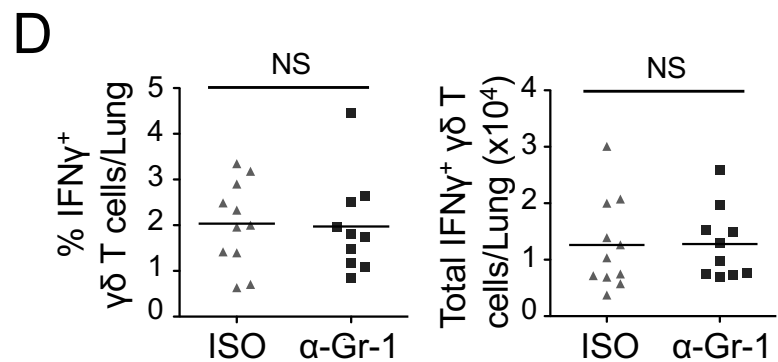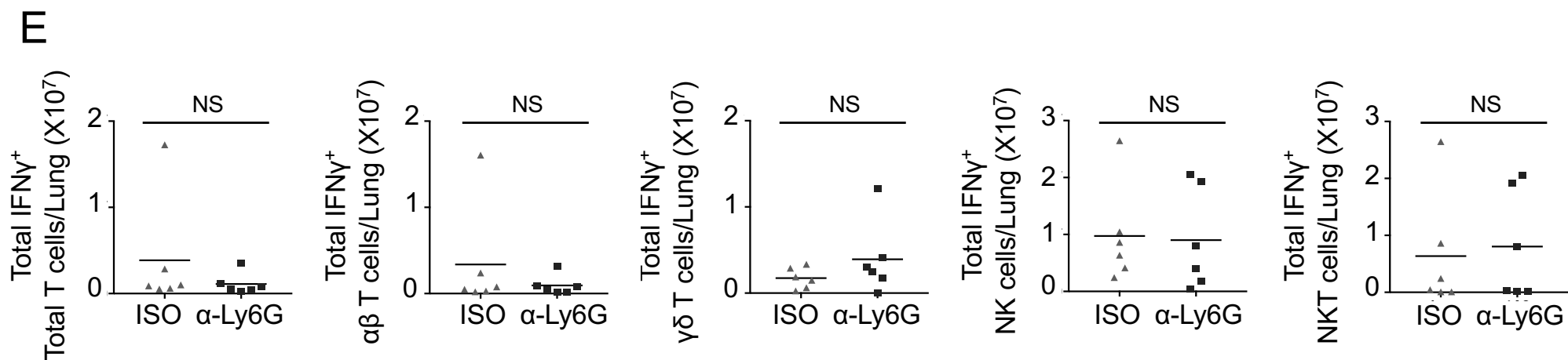

Supplement: S7 Fig — Representative flow cytometry plots and graphs showing the percentages and total numbers of IFNγ+ NKT cells (A and B) or IFNγ+ γδ T cells (C and D) in the lungs of uninfected (naïve) B6 mice or ΔflaA Lp-infected B6 mice treated with isotype control (ISO) or anti-Gr-1 (α-Gr-1) antibody at 24 hours post-infection. (E) Graphs showing the total numbers of IFNγ+ T cells, NK cells and NKT cells in the lungs of ΔflaA Lp-infected B6 mice treated with either isotype control antibody (ISO) or anti-Ly6G (α-Ly6G) antibody, as determined by flow cytometry. Data shown are the pooled results of 3 independent experiments with 3 or 4 mice per group per experiment (A-D) or 2 independent experiments with 3 mice per group per experiment (E). * is p<0.05 by unpaired t-test. NS is not significant. (PDF) [file ppat.1006309.s007.pdf]

A

B6 infected

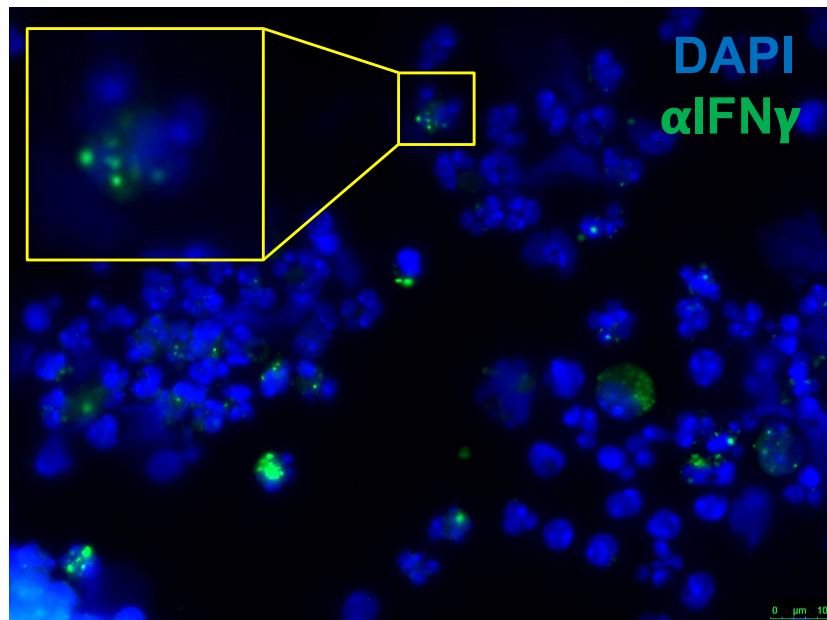

*Ifng*<sup>-/-</sup> infected

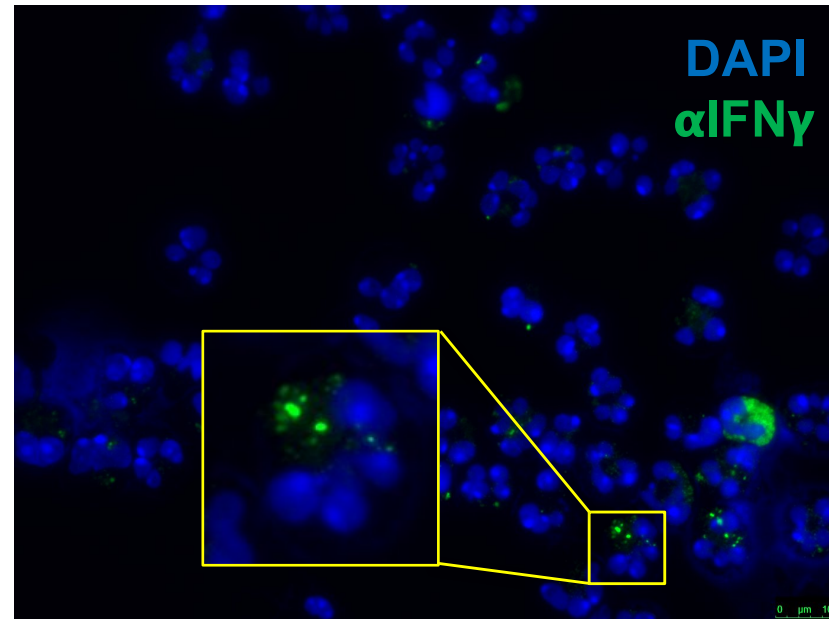

B

B6 infected

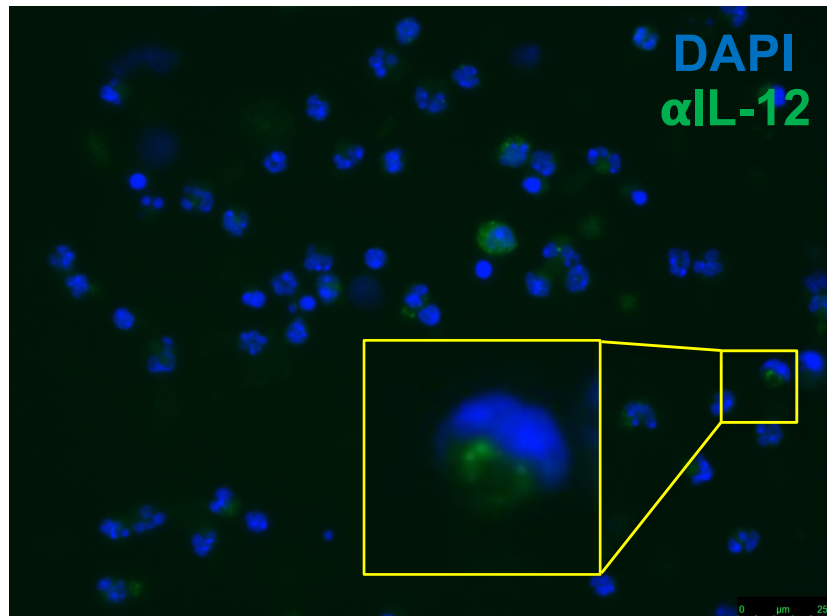

*Il12p40*<sup>-/-</sup> infected

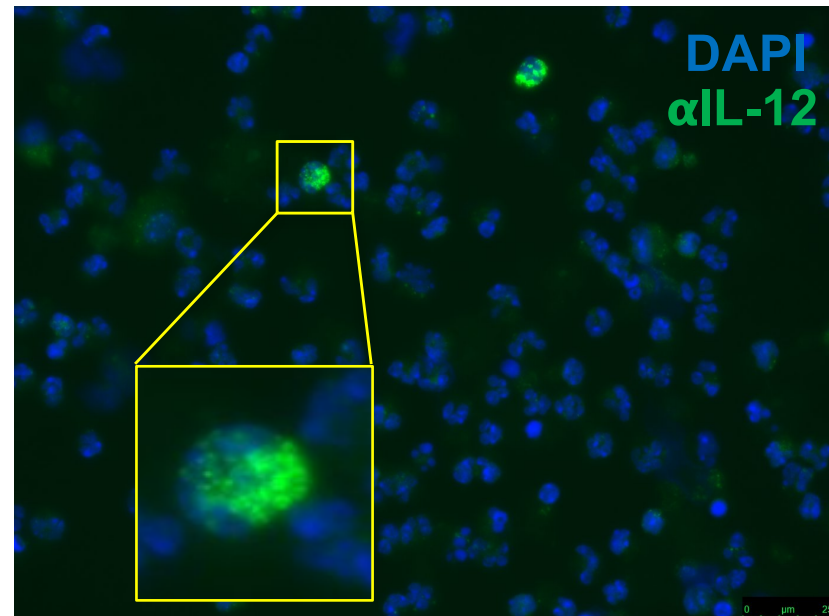

Supplement: S8 Fig — WT, Ifng-/- and Il12p40-/- mice were infected with ΔflaA L. pneumophila (Lp) and immunofluorescence microscopy analysis was performed on neutrophils harvested by BAL at 48 hours post-infection stained with anti-IFNγ or anti-IL-12 antibodies directly conjugated to AlexaFluor488. (A) Representative images of IFNγ immunofluorescence (40x). (B) Representative images of IL-12 immunofluorescence (20x). Shown are the merged DAPI and AlexaFluor488 channels. In each image, a representative cell with positive fluorescence signal is outlined in a yellow box and displayed in a magnified inset. (PDF) [file ppat.1006309.s008.pdf]
